# Blocking utilization of major plant biomass polysaccharides leads *Aspergillus* *niger* towards utilization of minor components

**DOI:** 10.1111/1751-7915.13835

**Published:** 2021-06-11

**Authors:** Roland S. Kun, Sandra Garrigues, Marcos Di Falco, Adrian Tsang, Ronald P. de Vries

**Affiliations:** ^1^ Fungal Physiology Westerdijk Fungal Biodiversity Institute & Fungal Molecular Physiology Utrecht University Uppsalalaan 8 Utrecht 3584 CT The Netherlands; ^2^ Centre for Structural and Functional Genomics Concordia University 7141 Sherbrooke Street West Montreal QC H4B 1R6 Canada

## Abstract

Fungi produce a wide range of enzymes that allow them to grow on diverse plant biomass. Wheat bran is a low‐cost substrate with high potential for biotechnological applications. It mainly contains cellulose and (arabino)xylan, as well as starch, proteins, lipids and lignin to a lesser extent. In this study, we dissected the regulatory network governing wheat bran degradation in *Aspergillus niger* to assess the relative contribution of the regulators to the utilization of this plant biomass substrate. Deletion of genes encoding transcription factors involved in (hemi‐)cellulose utilization (XlnR, AraR, ClrA and ClrB) individually and in combination significantly reduced production of polysaccharide‐degrading enzymes, but retained substantial growth on wheat bran. Proteomic analysis suggested the ability of *A. niger* to grow on other carbon components, such as starch, which was confirmed by the additional deletion of the amylolytic regulator AmyR. Growth was further reduced but not impaired, indicating that other minor components provide sufficient energy for residual growth, displaying the flexibility of *A. niger*, and likely other fungi, in carbon utilization. Better understanding of the complexity and flexibility of fungal regulatory networks will facilitate the generation of more efficient fungal cell factories that use plant biomass as a substrate.

## Introduction

Plant biomass is the most abundant carbon source on Earth and mainly consists of plant cell wall polymers (cellulose, hemicellulose, pectin and lignin) (de Vries and Visser, 2001). In nature, filamentous fungi, such as *Aspergillus niger*, secrete large arrays of hydrolytic enzymes to degrade the aforementioned polymers. Fungal Carbohydrate‐Active enZymes (CAZymes) are used in many industrial sectors for the production of pulp and paper, food and feed, detergents, textiles, and biofuels and biochemicals (Mäkelä *et al*., 2014). In this context, low‐cost substrates are of high interest for many biotechnological applications. Wheat bran, a by‐product of wheat milling, is the outer layer of wheat grain. It contains mainly cellulose and (arabino)xylan, as can be seen from the total sugar composition (Table S1). Wheat bran also contains starch, mixed‐linked β‐d‐glucans (including xyloglucan), as well as lignin, proteins and small amounts of lipids (DuPont and Selvendran, 1987; Stevens and Selvendran, 1988; Parker *et al*., 2005; Ruthes *et al*., 2017; Rudjito *et al*., 2019).

Transcriptional regulators or transcription factors play a key role in plant biomass degradation by fungi as they control the expression and synthesis of enzymes required for the degradation of different plant polysaccharides. The regulation system governed by transcription factors ensures that only those enzymes that are needed to degrade the prevalent substrate will be produced to avoid wasting energy on the production of enzymes that are not required. Several fungal transcription factors involved in plant biomass degradation have been identified across industrial species and fungal reference species (Benocci *et al*., 2017).

The first identified (hemi‐)cellulolytic regulator is the *A. niger* XlnR (van Peij *et al*., 1998b), and orthologs have been widely studied in other fungal species (Benocci *et al*., 2017), highlighting the key role of XlnR in the process of cellulose and hemicellulose utilization in fungi. Another hemicellulolytic transcription factor, AraR, controls the arabinanolytic system (Battaglia *et al*., 2011), and together with XlnR, controls the pentose catabolic pathway. The pentose catabolic pathway is required for the utilization of two main monomeric sugars found in hemicellulose, d‐xylose and l‐arabinose (Battaglia *et al*., 2014). In addition, the transcription factors Clr‐1 and Clr‐2 were identified in *Neurospora crassa*, and their corresponding gene deletions were shown to result in impaired cellulolytic activities (Coradetti *et al*., 2012). So far, Clr‐1 homologs showing conserved function have been reported for *A*. *nidulans* (Coradetti *et al*., 2012) and *A. niger* (ClrA) (Raulo *et al*., 2016) while Clr‐2 homologs have been described for multiple species, such as *A. nidulans* (Coradetti *et al*., 2013), *A. niger* (Raulo *et al*., 2016), *Penicillium oxalicum* (Yao *et al*., 2015) (ClrB) and *A*. *oryzae* (Ogawa *et al*., 2013). In *A. oryzae,* the Clr‐2 homolog was initially described as a regulator of β‐mannan utilization (ManR) (Ogawa *et al*., 2012), but whether it regulates mannan degradation in the other species has not been reported. In *A. niger*, ClrB plays a more dominant role in cellulose utilization compared with ClrA and both appear to be influenced by XlnR (Raulo *et al*., 2016). Considering the composition of wheat bran, these four transcription factors are expected to have a major role in its degradation by *A. niger*, while other transcription factors, such as AmyR (Petersen *et al*., 1999) (starch degradation), InuR (Yuan *et al*., 2008a) (inulin degradation) and GaaR (Alazi *et al*., 2016), RhaR (Gruben *et al*., 2014) and GalX (Kowalczyk *et al*., 2015) (pectin degradation), are expected to have no or a minor role.

In this study, we used CRISPR/Cas9 genome editing (Song *et al*., 2018) to generate a set of *A. niger* XlnR‐AraR‐ClrA‐ClrB deletion mutants to assess their relative contribution to wheat bran degradation and identify other possible transcription factors involved in wheat bran utilization by this fungus. The characterization of mutants carrying individual and combinatorial deletions of key transcription factors helps our understanding of the complexity of the regulatory network involved in the degradation of a crude substrate. This knowledge can facilitate the generation of fungal cell factories with high industrial applicability that use plant biomass as a substrate, through targeted engineering of the regulatory system.

## Results

### Combined deletion of *xlnR*, *araR*, *clrA* and *clrB* does not impair growth on wheat bran

**Fig. 1:**
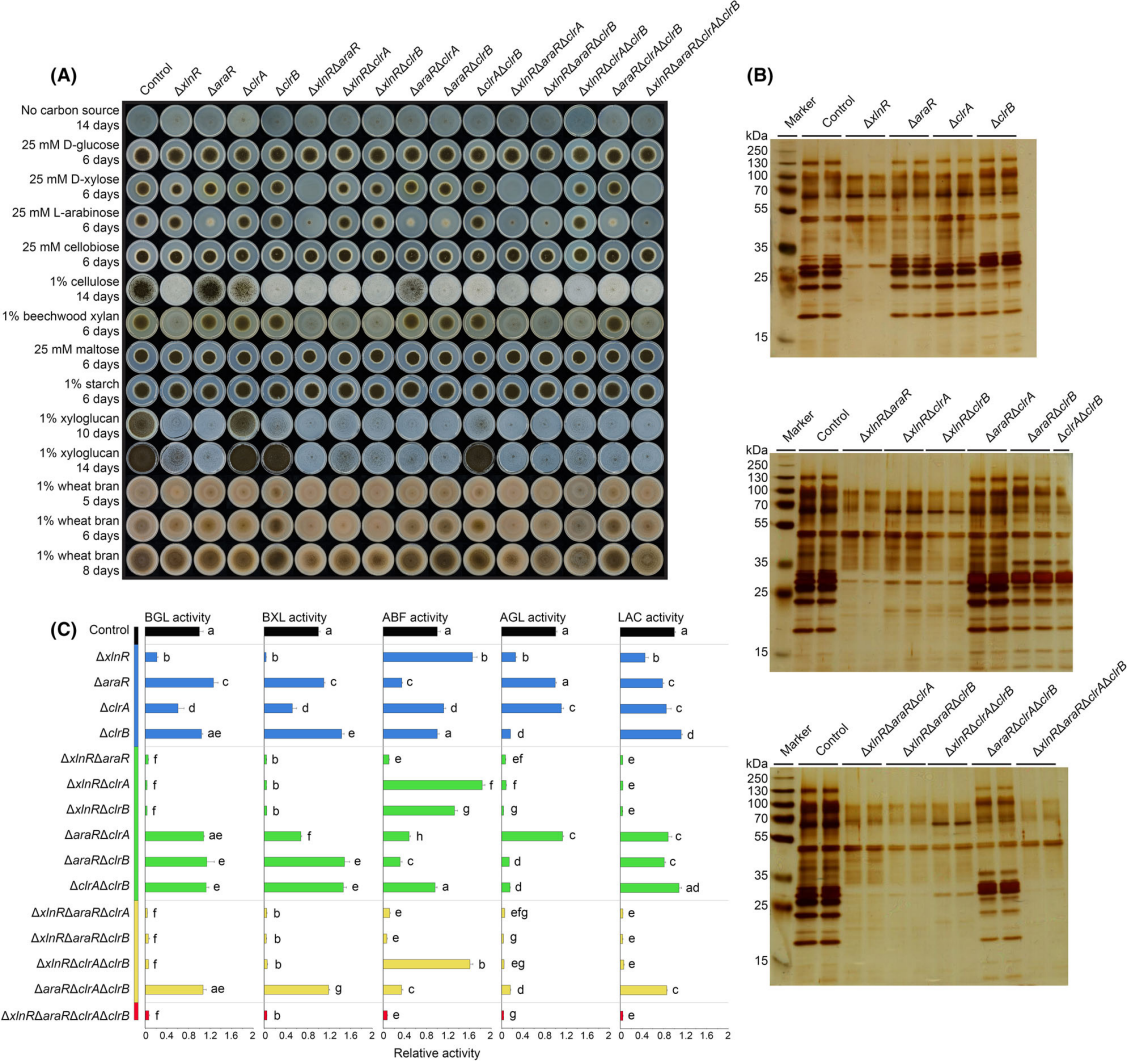
Characterization of *A. niger* XlnR‐AraR‐ClrA‐ClrB deletion mutants. A. Growth profile of *A. niger* control and mutant strains. Selected carbon sources were inoculated with 1000 spores and incubated at 30°C for up to 14 days. B. Extracellular protein production of *A. niger* control and mutant strains analysed by SDS‐PAGE after 24 h of growth on 3% wheat bran liquid cultures. Supernatant samples are analysed in biological duplicates. C. Enzyme activity assays of the supernatants from *A. niger* control and mutant strains. The control, and the single‐, double‐, triple‐ and quadruple mutant strains are indicated by different colours. Data represents the normalized mean values of biological duplicates and technical triplicates and the standard deviation. The absorbance values measured at 405 nm and the amount of released *p*‐nitrophenol by each strain are described in Data S1B. BGL = β‐1,4‐d‐glucosidase, BXL = β‐1,4‐xylosidase, ABF = α‐l‐arabinofuranosidase, AGL = α‐1,4‐d‐galactosidase, LAC = β‐1,4‐d‐galactosidase activity. Letters (a–h) are shown to explain the statistical differences between samples within each specific enzyme assay. Samples showing different letters show significant differences among the strains within each specific enzyme assay, while samples sharing the same letters show no statistically significant differences (ANOVA and Tukey's HDS test, *​P* < 0.05).

Null mutations of XlnR, AraR and ClrA reduced growth on wheat bran, while ClrB‐null resulted in improved growth on this substrate (Fig. 1A and Fig. S1). The improved growth of ClrB‐null was also observed in strains with combined deletions, but mainly when XlnR remained present in the strain. All other combined deletion strains showed reduced growth on wheat bran, but significant residual growth remained in all strains. To better capture the influence of the different regulators, growth was also evaluated on the polymeric and monomeric components of wheat bran (Fig. 1A). Deletion of *xlnR* or *clrB* abolished growth on cellulose, while Δ*clrA* and Δ*araR* mutants displayed reduced and normal growth, respectively. Only deletion of *xlnR* affected growth on xylan. While growth on xyloglucan was mostly affected by deletion of *araR* or *xlnR*, Δ*clrB* mutant only showed growth reduction at initial stages of growth on this substrate, and growth ability was recovered over time. No growth reduction was observed for any of the mutants on maltose, starch or cellobiose. Growth differences on d‐xylose and l‐arabinose reflect the influence of XlnR and AraR on sugar catabolism. Growth on d‐xylose was abolished in strains in which both *xlnR* and *araR* were deleted, but only minor differential growth phenotype compared with the control strain was observed in the other mutant strains. Growth on l‐arabinose was reduced in strains where *araR* was deleted, with a stronger reduction if *xlnR* was also absent.

Overall, the strongest growth reduction on wheat bran was observed in strains in which *xlnR* was deleted in combination with any of the other three transcription factors, especially after six days of growth. The Δ*xlnR*Δ*araR*Δ*clrA*Δ*clrB* mutant (subsequently referred to as quadruple mutant) was still able to grow on wheat bran, suggesting that it was utilizing other components, such as starch, a polysaccharide on which none of the tested mutants showed any differential phenotype.

### The relative growth reduction in the mutant strains correlates with reduced levels of key enzyme activities

To evaluate whether the reduced growth shown by the different mutant strains (Fig. 1A) could be a direct result of reduced enzyme levels, samples of liquid cultures containing 3% wheat bran were first analysed by SDS‐PAGE (Fig. 1B). Deletion of *xlnR* had the highest impact on the overall amount of extracellular protein, although in the quadruple mutant protein production was further reduced. Activity measurements for some relevant enzymes (Data S1A and B) were performed to provide insight into the molecular mechanisms that underlie the phenotypic differences between the strains.

The abolished growth of Δ*xlnR* on cellulose (Fig. 1A) correlates with the reduction in β‐1,4‐d‐glucosidase (BGL) activity in this strain (Fig. 1C), which is crucial for the release of d‐glucose units from cellulose or cellobiose. However, both growth and BGL activity suggest that ClrA is less involved in the regulation of cellulose degradation than XlnR in *A. niger*. The abolished growth of Δ*clrB* on cellulose cannot be explained by reduced BGL activity, suggesting that ClrB is more important for the production of cellobiohydrolases and/or endoglucanases (Raulo *et al*., 2016), which are crucial enzymes for degradation of cellulose.

d‐xylose release from (arabino)xylan, which is one of the most abundant polysaccharides of wheat bran, is catalysed by β‐xylosidase (BXL) activity. Similar to BGL activity, deletion of *xlnR* showed the highest impact on BXL activity (Fig. 1C), which was abolished in every mutant in which *xlnR* was deleted, and which correlated with minimal growth of these strains on beechwood xylan (Fig. 1A). Contrary to Δ*xlnR*, the Δ*araR* and Δ*clrB* mutants showed increased levels of BXL activity. However, they did not lead to increased growth on beechwood xylan. The BGL and BXL activities together with the growth profile indicate that XlnR has the overall highest impact on cellulose and (arabino)xylan utilization.

There was limited correlation between the levels of three accessory enzymes (α‐l‐arabinofuranosidase (ABF), α‐1,4‐d‐galactosidase (AGL) and β‐1,4‐d‐galactosidase (LAC), involved in degradation of several plant cell wall polysaccharides) (Fig. 1C) and the growth profile (Fig. 1A). However, the deletion mutants revealed which regulators affect the production of these enzymes. The Δ*clrA* mutant did not show decreased ABF or AGL activity while the reduction in LAC activity was minimal, indicating that ClrA does not control the genes encoding ABF, AGL or LAC. The Δ*xlnR* mutant showed significant reduction in AGL and LAC activities while deletion of *araR* significantly reduced ABF activity, and to a lesser extent LAC activity. Finally, deletion of *clrB* resulted in decreased AGL activity. Overall, the results confirm the regulatory roles of XlnR, AraR and ClrB on the genes encoding the enzymes responsible for the three accessory activities. Since these enzymatic activities are important for the degradation of xyloglucan, the results match the reduced ability of Δ*xlnR* and Δ*araR* to grow on xyloglucan. As ABF, AGL and LAC activities contribute to growth on xylan, the abolished enzymatic activities in the quadruple mutant in all tested conditions correlate with the abolished growth on cellulose, xylan and xyloglucan.

### Residual starch in wheat bran explains the limited growth reduction in the quadruple mutant

**Fig. 2:**
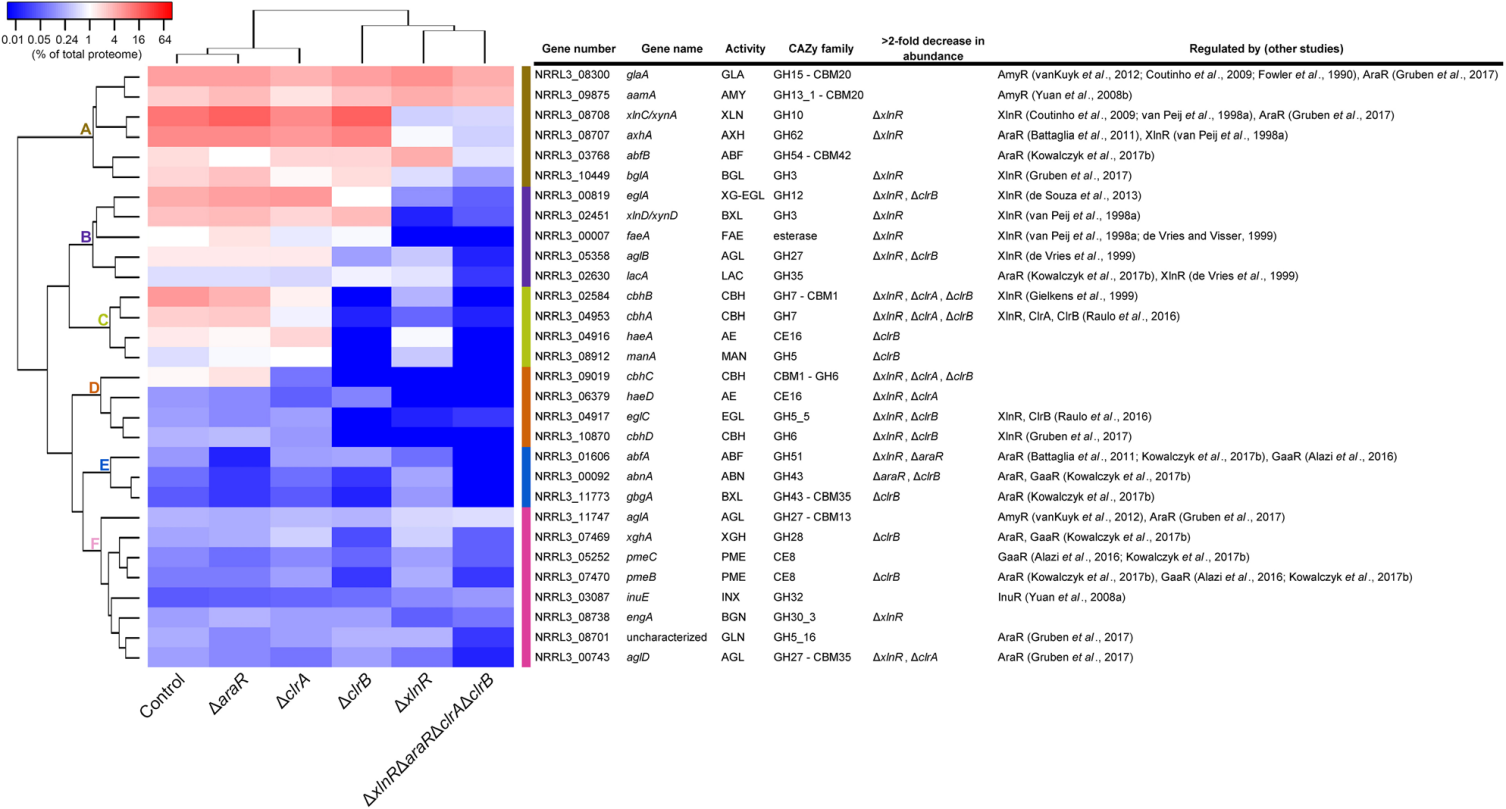
Hierarchical clustering of CAZymes found in the supernatant of *A. niger* control and Δ*xlnR*, Δ*araR*, Δ*clrA*, Δ*clrB* and Δ*xlnR*Δ*araR*Δ*clrA*Δ*clrB* mutant strains. The 24 h supernatant samples originated from 3% wheat bran liquid cultures. Colour code represents the averaged percentage value of the total exoproteome of duplicate samples. Regulation of genes is based on previous studies (Fowler *et al*., 1990; van Peij *et al*., 1998a; de Vries and Visser, 1999; de Vries *et al*., 1999; Gielkens *et al*., 1999; Yuan *et al*., 2008a, 2008b,2008a, 2008b; Coutinho *et al*., 2009; Battaglia *et al*., 2011; vanKuyk *et al*., 2012; de Souza *et al*., 2013; Alazi *et al*., 2016; Raulo *et al*., 2016; Gruben *et al*., 2017; Kowalczyk *et al*., 2017b). Enzyme abbreviations are described in Table S7. Enzymes with a lower abundance than 0.1% of the total proteome in each sample were excluded from the analysis. The complete exoproteome data are described in Data S2A.

Proteomics of selected samples (Data S2A) was performed to analyse in depth the effect of regulatory mutations on the production of individual plant biomass degrading enzymes in *A. niger*. The high amount of (arabino)xylan in wheat bran resulted in an abundant presence of xylanolytic enzymes (24.24% of the total exoproteome), as well as cellulolytic enzymes (four cellobiohydrolases and one BGL) in the control strain (Fig. 2 and Data S2B). However, the two detected endoglucanases represented only 0.16% (EglC) and 0.03% (EglB) (Data S2A) of the total exoproteome, correlating with the slow utilization of cellulose by *A. niger*.

Of the single deletion mutants, Δ*xlnR* showed the highest reduction in CAZymes, which is also reflected in the SDS‐PAGE (Fig. 1B) and enzyme activity (Fig. 1C) results. The minimal presence of (arabino)xylan‐acting enzymes in the Δ*xlnR* supernatant was similar to that of the quadruple mutant, although the overall abundance of cellulolytic enzymes was further reduced in the supernatant of the quadruple mutant (Fig. 2). Binding site analysis confirmed that all genes encoding the (arabino)xylanolytic and cellulolytic enzymes affected by the deletion of *xlnR* carry the putative XlnR binding site (GGCTAR) (de Vries *et al*., 2002) in their promoter sequences (Table S2), thus suggesting direct regulation of these genes by XlnR.

The quadruple deletion mutant showed further reduction in the abundance of two cellobiohydrolases (CbhA and CbhB), an acetylesterase (HaeA) and a β‐1,4‐endo‐mannanase (ManA) compared with the Δ*xlnR* mutant (Fig. 2, Cluster C). These are all enzymes that are negatively affected by the single *clrB* deletion, and the promoter sequences of their corresponding genes contain the putative ClrB binding site (CGGN_8_CCG) (Li *et al*., 2016) (Table S2), suggesting direct regulation of the Cluster C genes by ClrB. A broad range of other CAZymes showed highly reduced abundance or absence in the quadruple mutant compared with any single deletion strain (Fig. 2), suggesting combinatorial control by the studied transcription factors.

Amylases are the major carbohydrate‐degrading enzymes present in the secretome of the quadruple mutant (Data S2B). This correlates with the residual starch present in washed wheat bran (as described in Experimental procedures), which most likely has become the only carbohydrate that supports the growth of the quadruple mutant. Furthermore, nearly all proteins in the quadruple mutant decrease in abundance by at least twofold (Fig. 2). Among the few proteins that do not decrease in abundance in the quadruple mutant are three enzymes: a glucoamylase (GlaA), an α‐amylase (AamA) and an α‐1,4‐galactosidase (AglA). The genes encoding these enzymes have been shown to be regulated by the amylolytic transcription factor AmyR (Fowler *et al*., 1990; Yuan *et al*., 2008b; Coutinho *et al*., 2009; vanKuyk *et al*., 2012; Gruben *et al*., 2017).

**Fig. 3:**
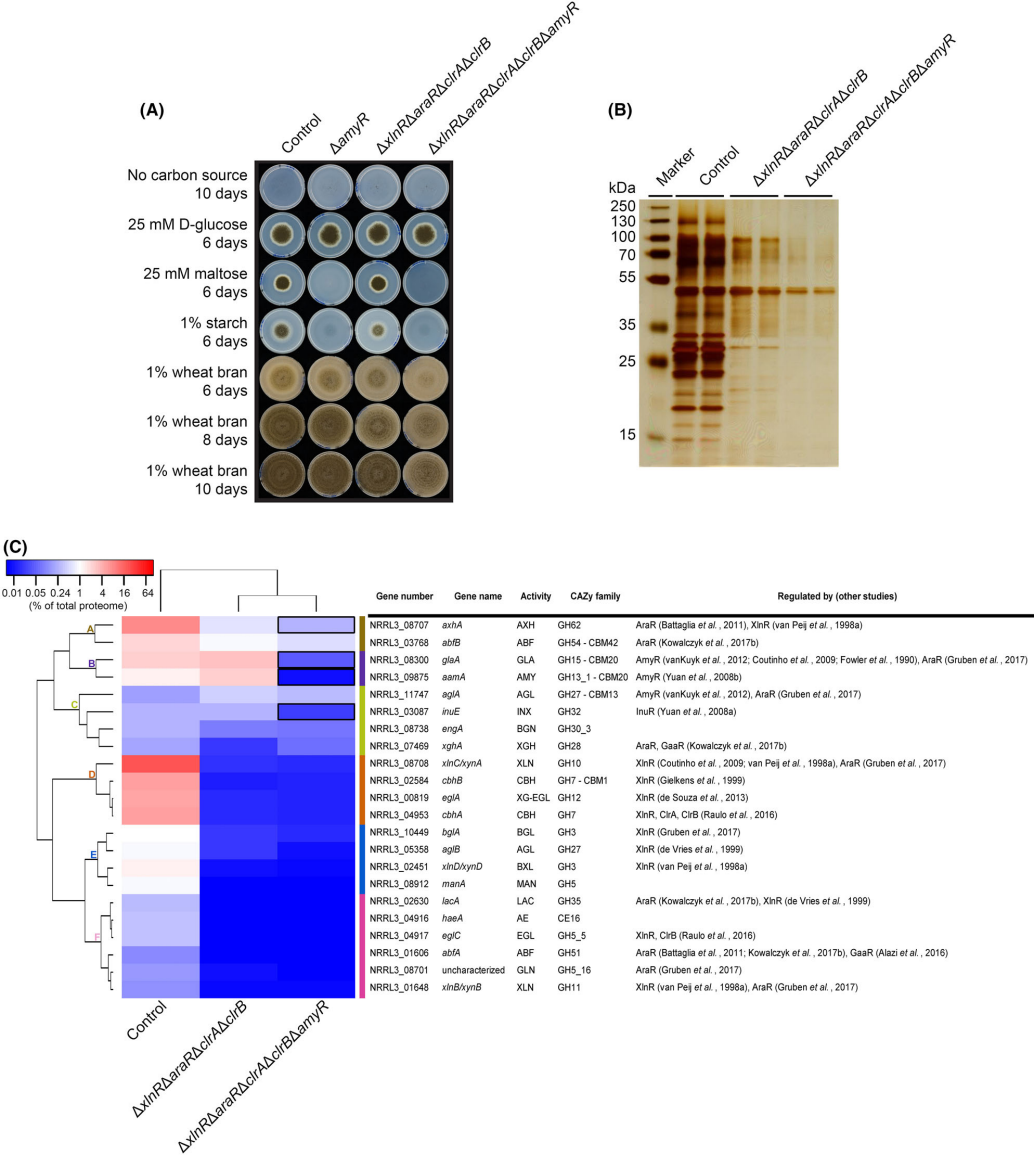
Characterization of *A. niger* Δ*xlnR*Δ*araR*Δ*clrA*Δ*clrB*Δ*amyR* quintuple deletion mutant. A. Growth profile of *A. niger* control, and the Δ*amyR*, Δ*xlnR*Δ*araR*Δ*clrA*Δ*clrB* and Δ*xlnR*Δ*araR*Δ*clrA*Δ*clrB*Δ*amyR* mutant strains. Selected carbon sources were inoculated with 1000 spores and incubated at 30°C for up to 10 days. B. Extracellular protein production of *A. niger* control and mutant strains analysed by SDS‐PAGE. The 24 h supernatant samples originated from 3% wheat bran liquid cultures. Samples are analysed in biological duplicates. C. Hierarchical clustering of CAZymes found in the supernatant of *A. niger* control, and the Δ*xlnR*Δ*araR*Δ*clrA*Δ*clrB* and Δ*xlnR*Δ*araR*Δ*clrA*Δ*clrB*Δ*amyR* mutant strains. Colour code represents averaged percentage value of the total exoproteome of duplicate samples. Black rectangles indicate CAZymes from the Δ*xlnR*Δ*araR*Δ*clrA*Δ*clrB*Δ*amyR* mutant showing > two fold decrease in abundance compared with the Δ*xlnR*Δ*araR*Δ*clrA*Δ*clrB* strain. Regulation of genes is based on previous studies (Fowler *et al*., 1990; van Peij *et al*., 1998a; de Vries *et al*., 1999; Gielkens *et al*., 1999; Yuan *et al*., 2008a, 2008b,2008a, 2008b; Coutinho *et al*., 2009; Battaglia *et al*., 2011; vanKuyk *et al*., 2012; de Souza *et al*., 2013; Alazi *et al*., 2016; Raulo *et al*., 2016; Gruben *et al*., 2017; Kowalczyk *et al*., 2017b). Enzyme abbreviations are described in Table S7. Enzymes with a lower abundance than 0.1% of the total proteome in each sample were excluded from the analysis. The complete exoproteome data are described in Data S3A.

To confirm the use of starch by the quadruple mutant during growth on wheat bran, we deleted *amyR* in this mutant and compared the phenotype to that of the quadruple mutant and the single Δ*amyR* strain. The Δ*amyR* mutant was unable to grow on maltose and starch, but growth on wheat bran was not affected after 6, 8 or 10 days (Fig. 3A). This unaltered growth suggests that the starch found in washed wheat bran contributes little to the growth of *A. niger*. In contrast, the Δ*xlnR*Δ*araR*Δ*clrA*Δ*clrB*Δ*amyR* mutant (subsequently referred to as quintuple mutant) showed a strong growth reduction compared with the quadruple mutant (Fig. S2). This result demonstrates the ability of *A. niger* to maintain growth by using the starch component of wheat bran, when utilization of the major carbohydrates is blocked.

### The Δ*xlnR*Δ*araR*Δ*clrA*Δ*clrB*Δ*amyR* mutant utilizes residual carbon sources found in wheat bran

Proteomics of liquid culture samples of the control, quadruple and quintuple mutant strains (Data S3A) demonstrated a further reduced protein production profile for the quintuple mutant compared with the quadruple strain due to the lack of amylolytic enzymes (Fig. 3B). The CAZyme content in the supernatant of the quintuple mutant (Fig. 3C) reduced to only 1.61% of the total exoproteome (Data S3B). The abundance of the single detected glucoamylase (GlaA) and the major α‐amylase (AamA) in the quadruple mutant was strongly reduced when *amyR* was deleted (Fig. 3C, cluster B). Moreover, the α‐1,4‐d‐glucosidase (AGD) and glucoamylase (GLA) activities involved in starch degradation have also been abolished in the quintuple mutant (Fig. S3 and Data S1C). These results suggest that the quintuple mutant is not able to utilize starch, which explains the observed further growth reduction on wheat bran (Fig. 3A). Interestingly, the abundance of an arabinoxylan arabinofuranohydrolase (AxhA) (Fig. 3C, cluster A) and an exo‐inulinase (InuE) (Fig. 3C, cluster C) was also reduced more than twofold compared with the quadruple strain. Analyses of the promoter sequences of their corresponding genes revealed that both carry putative AmyR binding sites (Table S2), suggesting that AmyR may be involved in the regulation of these genes.

**Fig. 4:**
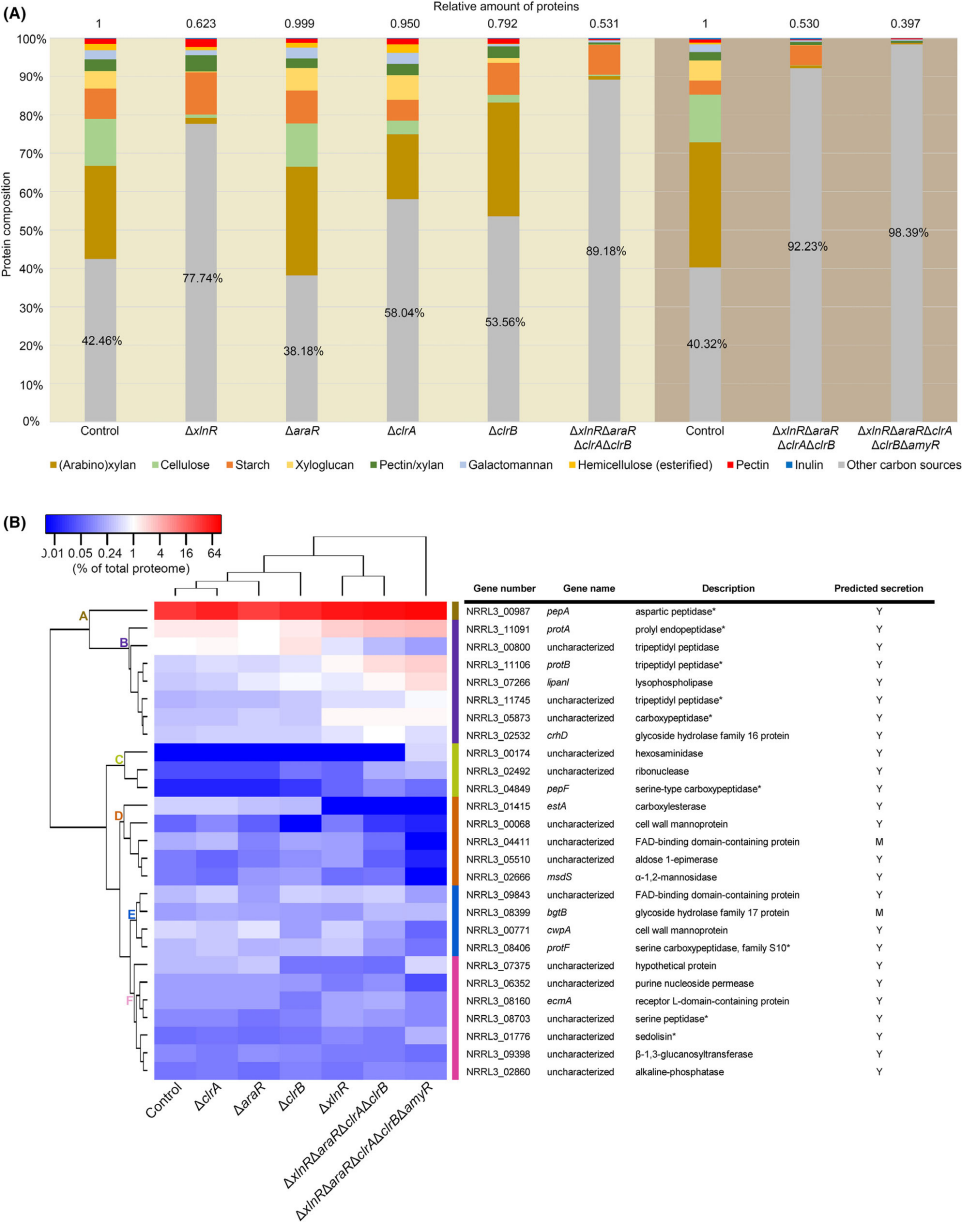
Analysis of non‐CAZyme proteins produced by *A. niger* control and deletion mutant strains. A. Relative composition of the total exoproteome of *A. niger* control and mutant strain supernatant samples originated from 3% wheat bran after 24 h. CAZymes are classified based on the substrates they are acting on and are indicated by different colours. Percentage values represent the abundance of non‐CAZymes indicated by grey colour. The relative amount of proteins produced by each culture indicated on top has been determined by RCDC kit assay after protein precipitation. The represented samples originated from two independent proteomic analyses (Data S2B and S3B) indicated by different background colours. B. Hierarchical clustering of non‐CAZymes found in the supernatant samples of *A. niger* control and mutant strains. The colour code represents the averaged percentage value of the total exoproteome of duplicates. Proteins under putative control of PrtT are indicated by (*). Y = predicted secretion; M = putative secretion. Proteins with abundance lower than 0.1% of the total proteome in each sample were excluded from the analysis. The control, Δ*xlnR*, Δ*araR*, Δ*clrA*, Δ*clrB* and Δ*xlnR*Δ*araR*Δ*clrA*Δ*clrB* represent the results from the first proteomic data set (Data S2A), while the Δ*xlnR*Δ*araR*Δ*clrA*Δ*clrB*Δ*amyR* data originate from the second proteomic data set (Data S3A).

The exoproteome of the quintuple mutant contained a broad range of proteases that were also observed in the other strains. Due to the reduction in CAZymes, the relative contribution of non‐CAZy proteins increased from 40.32% in the control strain to 98.39% in the quintuple strain (Fig. 4A and Data S3B). In all strains, the most abundant non‐CAZy protein was the aspartic peptidase PepA (Fig. 4B, cluster A), which is under the control of the transcription factor PrtT, a specific regulator of extracellular proteases in filamentous fungi (Punt *et al*., 2008). PepA, together with other PrtT‐controlled proteases (ProtA, ProtB, NRRL3_11745, PepF, NRRL3_05873 and NRRL3_01776) (Huang *et al*., 2020), showed a relative increase in abundance when the (hemi‐)cellulolytic enzyme system was impaired in the quadruple and quintuple deletion mutants (Fig. 4B and Data S2A and Data S3A). Lipases were detected in very small amounts, with only one (Lipanl) detected at > 0.1% abundance in all strains (Fig. 4B, cluster B), but with its highest abundance (1.95%) in the quintuple mutant (Data S3A). Overall, these results indicate that the quintuple mutant utilizes proteins as a primary carbon source when carbohydrate catabolism is blocked.

Predicted intracellular proteins are found in very low quantities in the secretome (Table S3), indicating that cell lysis did not occur extensively in our experiments. Moreover, the most abundant putative intracellular protein (NRRL3_00054) was also present in the control strain (Table S3), suggesting that the presence of some intracellular proteins may be linked to the experimental condition rather than to the deletion of transcription factors and poor growth. Overall, these results suggest that *A. niger* is able to survive on wheat bran after 24 h incubation by utilizing residual carbon sources when carbohydrate utilization is blocked.

## Discussion

The development of the biobased economy stimulates the development of fungal cell factories that convert plant biomass directly to desired products (e.g. proteins, metabolites) (Liaud *et al*., 2015; Amores *et al*., 2016). However, efficient design of such cell factories requires a detailed understanding of the plant biomass conversion process at the molecular level (Chroumpi *et al*., 2021). Plant biomass conversion by fungi involves a complex system of transcriptional regulation to ensure that the right set of enzymes is produced that matches the composition of the prevailing substrate. Several transcription factors involved in this process have been identified in fungi, but their relative contribution, interaction and possible overlapping sets of target genes have not been addressed in detail. In the present study, we addressed these questions by performing an in‐depth analysis of the contribution of five transcriptional activators involved in the conversion of wheat bran by *A. niger*.

Exoproteomics of the control strain revealed that *A. niger* degrades mainly (arabino)xylan. However, enzymes involved in cellulose degradation do not appear to be coordinately regulated under growth on wheat bran. Exo‐acting cellulases are abundantly represented in the exoproteome while endoglucanases, which are essential for efficient degradation of cellulose, are detected at low levels. The low abundance of endoglucanases together with the slow growth on cellulose suggests that cellulose is not a preferred carbon source for *A. niger*. Lower levels of amylolytic and xyloglucanolytic enzymes were also detected, which correlates with the levels of xyloglucan and residual starch reported to be present in washed wheat bran (DuPont and Selvendran, 1987; Nyombaire, 2012). In addition, mannanolytic, pectinolytic and inulinolytic enzymes were detected, but based on the composition of wheat bran, it is not likely that galactomannan, pectin and inulin are present in sufficient amounts to support growth of *A. niger*. Nevertheless, trace amounts of the inducers for the production of these enzymes may explain their presence, as it was observed previously in a transcriptome study of *A. niger* during growth on guar gum (Coconi‐Linares *et al*., 2019). Although the composition of fungal CAZymes is largely dependent on the incubation time and substrate composition, comparable results have been reported for the thermophilic fungus *Myceliophthora thermophila*. A combined transcriptome and exoproteome study showed mainly the upregulation of genes involved in xylan and cellulose degradation when grown on monocot plants (Kolbusz *et al*., 2014). However, a comparative study of the *Trichoderma reesei* and *Talaromyces cellulolyticus* (formerly *Acremonium cellulolyticus*) secretomes showed differences in the major enzymatic activities (Fujii *et al*., 2009). The supernatant derived from *T. cellulolyticus* showed higher cellulolytic activity and d‐glucose yield from plant biomass, while the *T. reesei* supernatant showed higher xylanolytic activities than the supernatant of *T. cellulolyticus*. The synergistic action of xylanases and cellulases is necessary for the enzymatic degradation of various agricultural residues or woody substrates for biofuel production (Álvarez *et al*., 2016).

The phenotype and exoproteome of the *A. niger* Δ*xlnR* mutant confirmed previous studies that reported impaired growth on cellulose, (arabino)xylan and xyloglucan (van Peij *et al*., 1998a; Gruben *et al*., 2017). Enzyme activity assays and proteomic studies also demonstrate the key role of XlnR in the utilization of (arabino)xylan and cellulose when grown on wheat bran, and correlate with a previous study that showed that colonization of wheat bran is mainly dependent on XlnR (Kowalczyk *et al*., 2017a). However, it cannot be excluded that the phenotype of Δ*xlnR* includes reduced production of ClrA and ClrB targets, as it has previously been shown that XlnR affects the expression of the genes encoding these two transcription factors (Raulo *et al*., 2016). Therefore, we conclude that XlnR is the dominant transcription factor for wheat bran utilization by *A. niger*.

Deletion of *clrA* has low impact on growth on the tested substrates. The significantly reduced BGL and BXL activity in the single Δ*clrA* mutant suggests that ClrA at least in part regulates the expression of their encoding genes. However, no reduced BGL activity was observed in the Δ*araR*Δ*clrA*, Δ*clrA*Δ*clrB* and Δ*araR*Δ*clrA*Δ*clrB*, while BXL activity was increased in the Δ*clrA*Δ*clrB* and Δ*araR*Δ*clrA*Δ*clrB* mutants. We therefore conclude that BGL and BXL encoding genes are controlled by XlnR and ClrA in *A. niger*. Furthermore, our proteomic results show that the main BGL (BglA) and BXL (XlnD/XynD) proteins are controlled by XlnR and ClrA, and XlnR appears to be able to compensate for the loss of *clrA* in the absence of *araR* and/or *clrB*. These results suggest that under our conditions, ClrA is not crucial for the regulation of BGL and BXL activities, but is part of the interactive regulatory system during growth of *A. niger* on wheat bran.

ClrB has been previously described to have a more extensive role in the breakdown of wheat straw compared with ClrA in *A. niger* (Raulo *et al*., 2016), in particular with respect to cellulose utilization. Our proteomics and enzyme assay data suggest that this transcription factor controls the expression of cellobiohydrolases and endoglucanases, but not BGL genes, which is supported by the presence of a putative ClrB binding site (Li *et al*., 2016) in the promoters of endoglucanases and cellobiohydrolases, but not in the analysed BGL gene (*bglA*). The impaired cellobiohydrolase and endoglucanase production shown by the *clrB* deletion mutant correlates with its inability to grow on cellulose.

The growth profile, enzyme activity assays and exoproteomics of the single Δ*araR* mutant suggest a minor role for AraR in the degradation of wheat bran. Deletion of *araR* only abolished growth on xyloglucan, which is a minor component of wheat bran. The abolished growth can possibly be explained by the reduced ability of Δ*araR* to remove l‐arabinose units from the sidechains decorating xyloglucan, most likely mediated by AbfB (de Vries and Visser, 2001). This observation correlates with our results, showing highly reduced ABF activity, as well as reduced abundance of both analysed ABFs (AbfA and AbfB) in the exoproteome of Δ*araR* strain. All the genes encoding these proteins have been previously described to be under the control of AraR (Battaglia *et al*., 2011).

In general, results from growth profiling and enzyme assays show that gene co‐regulation is required for efficient utilization of crude substrates. Gene co‐regulation by three different transcription factors (AraR, GaaR, RhaR) has already been reported in the case of sugar beet pectin degradation in *A. niger* (Kowalczyk *et al*., 2017b). In our case, none of the single (hemi‐)cellulolytic transcription factor deletion mutants showed strong reduction in growth on wheat bran, but a small reduction was observed in the quadruple mutant, indicating the integrative roles of the studied transcription factors in the overall utilization of this crude substrate. In addition, alternative carbon components of wheat bran can still largely compensate for the inability to use (hemi‐)cellulose, as evidenced by the abundant growth of the quadruple mutant on wheat bran. The high abundance of CAZymes involved in starch utilization in the quadruple mutant's exoproteome suggested that utilization of starch was responsible for growth. Degradation of starch is mainly controlled by AmyR (Gomi *et al*., 2000). The strong reduction in growth of the quintuple mutant indicated that starch is responsible for the only small growth reduction in the quadruple mutant, a phenotype confirmed by the exoproteome data of the quintuple mutant, where the major amylolytic enzymes (GlaA and AamA) were strongly reduced. Moreover, the deletion of *amyR* also resulted in the decreased abundance of the arabinoxylan arabinofuranohydrolase AxhA and the exo‐inulinase InuE in the quintuple mutant. These results suggest that the role of AmyR extends beyond starch degradation, as it was also shown to control the expression of BGL, AGL and LAC encoding genes (vanKuyk *et al*., 2012). However, the quintuple mutant still showed residual growth on wheat bran, most likely by utilizing proteins and other minor components, such as lipids (Morrison, 1978; Benoit *et al*., 2015). The presence of these components is supported by the slightly upregulated protease profile of the quintuple mutant and the presence of lipases in the exoproteome. The most abundant proteases found in the exoproteome are controlled by PrtT (Punt *et al*., 2008; Huang *et al*., 2020). The major PrtT‐controlled proteases (PepA, ProtA and ProtB) represented a relatively higher proportion of the exoproteome in the quintuple mutant compared with the quadruple deletion strain. This may be caused by the deletion of AmyR, as AmyR has been shown to have a negative effect on PrtT‐mediated regulation of protease gene expression (Huang *et al*., 2020). However, it is more likely that this is a starvation‐induced response as utilization of the major carbohydrates is blocked in the quintuple mutant and starvation has been shown to cause protease induction (Nitsche *et al*., 2012). The adaptation to the utilization of proteins in the quintuple mutant could be further investigated by the additional deletion of PrtT. This approach could possibly result in the abolishment of the residual growth that we observed on wheat bran. Finally, our data also show that while the quintuple mutant grows slower, it is apparently still highly viable as cell lysis did not occur extensively in this mutant, as indicated by a low total abundance of putative intracellular proteins. This supports our hypothesis that *A. niger* is able to utilize proteins and other minor non‐carbohydrate components present in wheat bran when the utilization of every major and even residual carbohydrates is blocked in this species.

To our knowledge, this is the first study in which the major transcription factors involved in the regulation of CAZymes required for the utilization of the major (and most abundant) polysaccharides found in a crude substrate have been studied in combination in a fungus. Using combinatorial deletions, we achieved a strain showing a minimal CAZyme content in its extracellular proteome, making it unlikely for the fungus to be able to utilize any carbohydrates found in wheat bran. By analysing the single deletion mutants, we observed an unexpected growth improvement on wheat bran for the *clrB* deletion mutant, which correlated with an increased abundance of the main xylanolytic enzymes and improved BXL activity. This may be the result of a (in)direct interaction with XlnR, through a currently unknown mechanism. Moreover, we observed a transient growth of Δ*clrB* and an abolished growth of Δ*araR* mutant on xyloglucan. The deficient growth may correlate with the overall decreased abundance of xyloglucanases and α‐l‐arabinofuranosidases in the Δ*clrB* and Δ*araR* strains, respectively. These results provide leads for additional studies into the interaction between individual transcription factors.

**Fig. 5:**
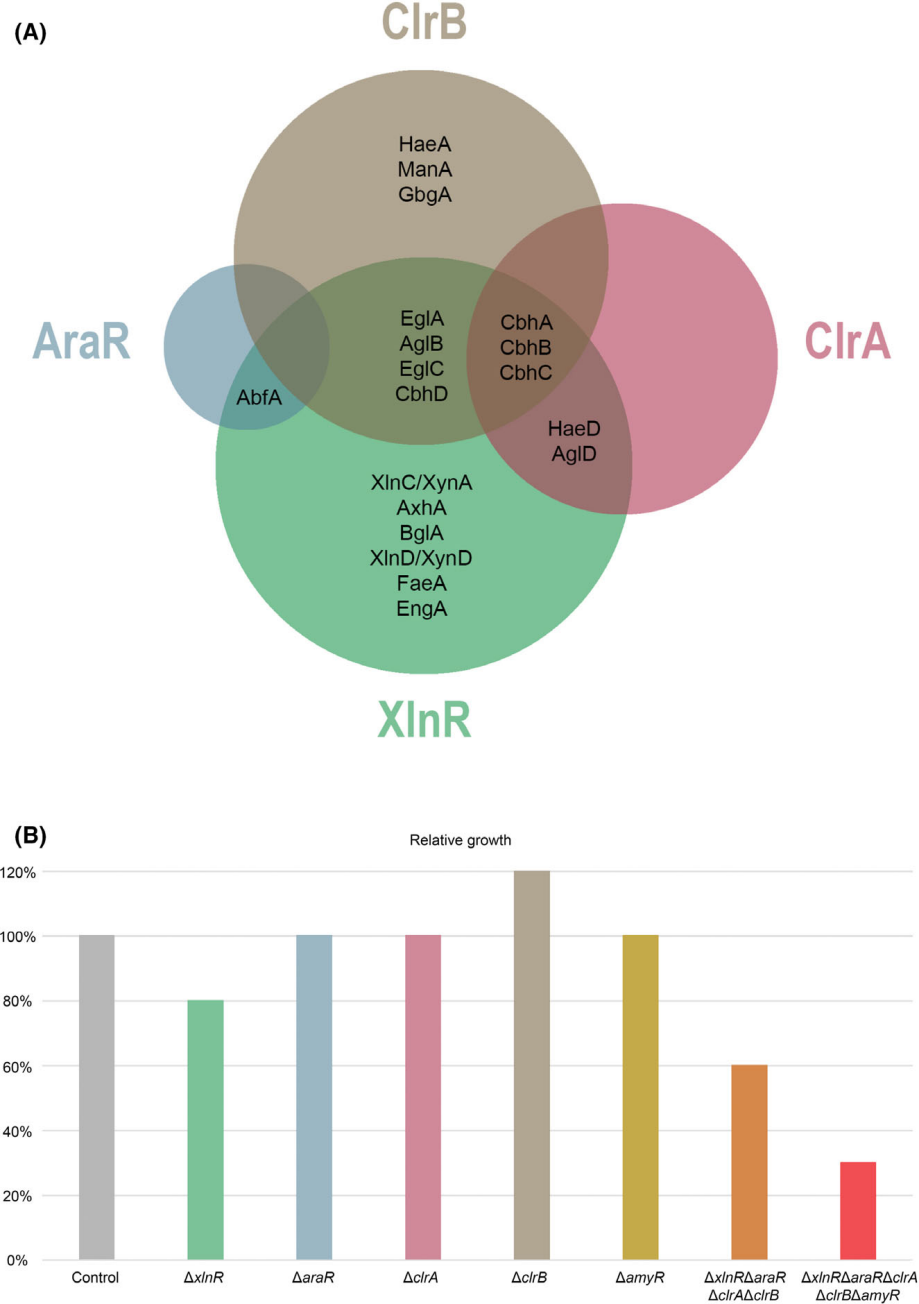
Hierarchy of transcriptional activators involved in wheat bran utilization. A. Contribution of XlnR, AraR, ClrA and ClrB in the regulation of major (hemi‐)cellulases when grown on wheat bran. The (hemi‐)cellulose‐specific CAZymes that showed >two fold decrease in abundance in the exoproteome of single deletion mutants (Data S2A) are indicated under the control of the corresponding transcription factor. The regulated enzymes include one α‐l‐arabinofuranosidase (AbfA), one β‐d‐arabinoxylan arabinofuranohydrolase (AxhA), two acetyl esterases (HaeA and HaeD), one feruloyl esterase (FaeA), two α‐1,4‐galactosidases (AglB and AglD), one β‐1,4‐endo‐mannanase (ManA), one β‐1,6‐endoglucanase (EngA), one β‐1,4‐endoglucanase (EglC), four cellobiohydrolases (CbhA, CbhB, CbhC and CbhD), one β‐1,4‐glucosidase (BglA), one xyloglucanase (EglA), one β‐1,4‐endo‐xylanase (XlnC/XynA) and two β‐1,4‐xylosidases (XlnD/XynD and GbgA). B. Relative contribution of XlnR, AraR, ClrA, ClrB and AmyR towards utilization of wheat bran. Contribution of each transcription factor is represented by the relative growth reduction in the corresponding deletion mutants compared with the control. Relative growth has been estimated after 6 days of incubation at 30°C. No growth difference was observed between biological replicates.

In conclusion, our study shows hierarchical roles of the studied transcription factors with respect to (hemi‐)cellulose utilization in wheat bran. XlnR is the major transcription factor for this substrate in *A. niger*, followed by ClrB, while ClrA and AraR show lower contribution (Fig. 5A). The apparent minor role of AraR, ClrA and ClrB in wheat bran utilization is most likely due to their overall low contribution to (arabino)xylan degradation. We also conclude that AmyR contributes to the degradation of wheat bran components (Fig. 5B). The hierarchy of all these transcriptional activators with respect to their relevance for wheat bran degradation may be species dependent and therefore explain the diverse enzyme sets published for different species during growth on wheat bran or other plant biomass substrates (Benoit *et al*., 2015; de Vries *et al*., 2017). This hypothesis is supported by the different roles of ClrA and ClrB in *N. crassa* (Coradetti *et al*., 2012), *A. nidulans* (Coradetti *et al*., 2012) and *A. oryzae* (Ogawa *et al*., 2013), compared to *A. niger*, as well as the highly diverse set of target genes of XlnR in different fungi (Klaubauf *et al*., 2014). Finally, we show that *A. niger* prefers to utilize (arabino)xylan over cellulose, but it is also able to maintain growth through the utilization of residual polysaccharides or even proteins and other minor components when the utilization of the main polysaccharides is blocked. These results highlight that *A. niger* possesses a flexible regulatory system, facilitating the use of most of the components found in plant biomass, which is likely a major reason for its high suitability for industrial applications.

## Experimental procedures

### Strains, media and growth conditions

*Escherichia coli* DH5α was used for plasmid propagation and was grown in Luria‐Bertani (LB) medium supplemented with 50 μg ml^−1^ ampicillin (Sigma‐Aldrich). Fungal strains used in this study were derived from the *A. niger* CBS 138852 (*cspA1*, *pyrG^−^*, *kusA*::*amdS*) strain (Meyer *et al*., 2007). The generated mutants were deposited at the culture collection of Westerdijk Fungal Biodiversity Institute under accession numbers indicated in Table S4. Fungal strains were grown at 30°C on *Aspergillus* Minimal Medium (MM) or Complete Medium (CM) (de Vries *et al*., 2004) supplemented with 1% d‐glucose and 1.22 g l^−1^ uridine (Sigma‐Aldrich).

Growth profiles were performed using *Aspergillus* MM containing 25 mM d‐glucose/d‐xylose/l‐arabinose/maltose (Sigma‐Aldrich), 25 mM cellobiose (Acros Organics) or 1% cellulose/beechwood xylan/xyloglucan/starch/wheat bran. The wheat bran used in this study was washed to remove free monosaccharides and a large part of the soluble starch (Nyombaire, 2012). Washing was performed by autoclaving wheat bran at 5% concentration in demineralized Milli‐Q water. After autoclaving, the medium was centrifuged at 1800 × *g* for 10 min. The supernatant was removed and the insoluble wheat bran pellet was resuspended in sterile demineralized Milli‐Q water. The suspension was centrifuged again, and the final supernatant‐free pellet was resuspended in MM with 1% final concentration for growth profile or 3% final concentration for liquid cultures. All media were supplemented with 1.22 g l^−1^ uridine. All growth profile plates were inoculated in duplicates with 1000 spores and incubated at 30°C for up to 14 days. Pictures were taken after 5, 6, 8, 10 and 14 days of incubation and evaluated by visual inspection, taking into account colony diameter, mycelial density and sporulation.

For liquid cultures, freshly harvested spores were pre‐grown in 250 ml CM containing 2% d‐fructose and 1.22 g l^−1^ uridine for 16 h at 30°C in a rotary shaker at 250 rpm. After 16 h, mycelia were harvested by filtration through sterile cheesecloth, rinsed with MM, and approximately 2.5 g (wet weight) of mycelium were transferred into 50 ml MM containing 3% wheat bran. Supernatant samples were taken after 24 h of incubation at 30°C in a rotary shaker at 250 rpm. The samples were centrifuged (20 min, 3220 × *g*, 4°C), and cell‐free supernatant samples were stored at −20°C until further processing.

### Construction of mutant strains

The ANEp8‐Cas9‐*pyrG* plasmid (Song *et al*., 2018), which contains the autonomous fungal replicating element AMA1 (Gems *et al*., 1991), *pyrG* as selection marker, *cas9* gene and the guide RNA (gRNA) expression construct under the control of the proline transfer ribonucleic acid (tRNA^Pro1^) promoter, was used in this study.

Selection of guide RNA (gRNA) sequences was performed using the Geneious 11.1.4 software (https://www.geneious.com) based on the methodology described by Doench and collaborators (Doench *et al*., 2014). Repair templates, which include the 5′ and 3′ flanking regions of the target genes, were amplified and fused together using fusion‐PCR. Flanking regions represent 500–1000 bp homologous sequences before and after the target gene's open reading frame (ORF).

CRISPR/Cas9 plasmid construction, generation of *A. niger* protoplasts, transformation and purification of putative mutant strains was performed as previously described (Kun *et al*., 2020). The Δ*xlnR*, Δ*araR*, Δ*xlnR*Δ*araR*, as well as the Δ*clrA*, Δ*clrB* and Δ*clrA*Δ*clrB* mutants were obtained by simultaneous double deletions using the *A. niger* CBS 138852 strain as background. The Δ*xlnR*, Δ*araR* and Δ*xlnR*Δ*araR* mutant strains have been used as background for further deletion of Δ*clrA* and Δ*clrB*, resulting in all possible combinations of deletions. Finally, the *amyR* gene was deleted in the Δ*xlnR*Δ*araR*Δ*clrA*Δ*clrB* strain by performing a single deletion.

Mutant strains have been confirmed by analytical PCR, through the amplification of the target gene region. All primers used in this study were ordered from Integrated DNA Technologies (IDT, Leuven, Belgium) and are shown in Table S5.

### SDS‐PAGE and enzyme activity assays

Cell‐free supernatant samples of 3% wheat bran liquid cultures were harvested after 24 h of incubation at 30°C in a rotary shaker at 250 rpm. Twelve microlitres of supernatant samples have been mixed with 4 μl loading buffer (10% of 1 M Tris‐HCl, pH 6.8; 42% glycerol, 4% (w/v) SDS; 0.02% (w/v) bromophenol blue; 4% of 14.7 M mercaptoethanol), of which 10 μl aliquots have been analysed by SDS‐PAGE as previously described (Kun *et al*., 2020). Enzyme activities were evaluated based on colorimetric *p*‐nitrophenol (pNP) assays. Supernatant samples (10 μl) were mixed with 10 μl 0.1% 4‐nitrophenyl β‐d‐glucopyranoside (for BGL activity), 0.1% 4‐nitrophenyl β‐d‐xylopyranoside (for BXL activity), 0.1% 4‐nitrophenyl α‐l‐arabinofuranoside (for ABF activity), 0.1% 4‐nitrophenyl α‐d‐galactopyranoside (for AGL activity), 0.1% 4‐nitrophenyl β‐d‐galactopyranoside (for LAC activity), 0.1% 4‐nitrophenyl α‐d‐glucopyranoside (for AGD activity) or 0.1% 4‐nitrophenyl maltoside (for GLA activity) substrates, 50 μl 50 mM NaAc (pH 5) and 30 μl demineralized water in a final volume of 100 μl. BGL, BXL and LAC activities were measured after 1 h, ABF activity was measured after 30 min, and AGL activity was measured after 15 min, while AGD and GLA activities were measured after 20 h of incubation at 30°C. The reactions were stopped by the addition of 100 μl of 0.25 M Na_2_CO_3_, and absorption values were measured at 405 nm wavelength using FLUOstar OPTIMA (BMG Labtech). All measurements were performed by using biological duplicates and technical triplicates.

### Statistical analysis

The number of experimental replicates is described in the figure legends. Differences in enzyme activities were determined using the one‐way analysis of variance (ANOVA) and Tukey’s HSD test (Table S6). Statistical significance was referred for *p* value < 0.05. Analyses were done using Statgraphics Centurion XVI Version 16.1.17 (www.statgraphics.com/centurion‐xvi).

### Proteomic analysis

Proteins from 500 μl cell‐free supernatant aliquots were precipitated by mixing them with two volumes of −20°C methanol, followed by overnight incubation at −20°C. The protein solution was centrifuged at 20800 × *g*, 4°C for 20 min. The supernatant was aspired, and the pellet was washed with 60% cold methanol solution and was resuspended in a 6 M urea, 100 mM ammonium bicarbonate pH 8 solution. Protein amounts have been determined colorimetrically by using the RCDC kit assay (Bio‐Rad, Mississauga, ON, Canada). Five micrograms of protein samples of biological duplicates were digested with trypsin for proteomic analysis as previously described (Budak *et al*., 2014). Dried peptide digest samples were solubilized in a solution of 5% acetonitrile, 0.1% formic acid and 4 fmol µl^−1^ of trypsin‐digested bovine serum albumin (BSA) (Michrom, Auburn, CA) used as internal standard. Five microlitres were analysed by LC‐MS/MS using an Easy‐LC II Nano‐HPLC system connected in‐line with a Velos LTQ‐Orbitrap mass spectrometer (Thermo Fisher, San Jose, CA). LC‐MS/MS data peptide and protein identification were done using the *A*. *niger* NRRL3 protein sequence databases. Protein identification and quantification were performed using the Proteome Discoverer 2.2 (Thermo Fisher) precursor ion quantitation workflow. Normalized individual protein area values were expressed as a fold value of the protein area value determined for the BSA internal standard. The abundance of proteins has been analysed using percentage values of the total exoproteome.

Heat maps for proteome data visualization were generated using the ‘gplots’ package of r software, with the default parameters: ‘Complete‐linkage clustering method and Euclidean distance’. Proteins with a lower abundance than 0.1% of the total proteome in each sample were excluded from the analysis.

### Binding site analysis

Binding site analysis for the target transcription factors was performed using the RSAT online tool (Thomas‐Chollier *et al*., 2008) (http://rsat‐tagc.univ‐mrs.fr/rsat/dna‐pattern_form.cgi). The 1000 bp length promoter sequences upstream of the coding regions of the analysed genes were obtained from the JGI MycoCosm database (https://genome.jgi.doe.gov/Aspni_NRRL3_1/Aspni_NRRL3_1.home.html). Binding sites were searched using the ‘DNA Pattern Matching’ algorithm, with the default parameters of ‘search on both strands’ and ‘prevent overlapping matches’. The reported putative binding motifs 5′‐GGCTAR‐3′ (de Vries *et al*., 2002) and 5′‐CGGNTAAW‐3′ (Ishikawa *et al*., 2018) for XlnR, 5′‐CGGDTAAW‐3′ (Ishikawa *et al*., 2018) for AraR, 5′‐CGGN_8_CCG‐3′ (Li *et al*., 2016) for ClrB and 5′‐CGGN_8_CGG‐3′ (Petersen *et al*., 1999) for AmyR were analysed in this study.

## Conflict of interest

The authors declare no conflict of interest.

## Author contributions

R.S.K. performed the experiments, analysed data and wrote the original manuscript. S.G. contributed to data analysis and manuscript writing. M.D.F. performed the proteomic analysis. A.T. and R.P.dV. designed the experiments, supervised the research, reviewed and edited the manuscript.

## Supporting information


**Data S1**. Enzymatic activities measured in this study. (A) Enzymatic activities and their substrates. The substrates present in wheat bran are highlighted in bold. (B) Enzyme assay results. Statistical analysis was performed using the converted (nmol/min/ml) values, while the visualization (Fig. 1C) was performed using the normalized mean and standard deviation (SD) values. (C) α‐glucosidase (AGD) and glucoamylase (GLA) activity assay results. Statistical analysis was performed using the converted (nmol/min/ml) values, while the visualization (Fig. S3) was performed using the normalized mean and standard deviation (SD) values

**Data S2**. Extracellular proteome of *A. niger* control and Δ*xlnR*, Δ*araR*, Δ*clrA*, Δ*clrB* and Δ*xlnR*Δ*araR*Δ*clrA*Δ*clrB* mutant strains. (A) Proteomics results of *A. niger* control and Δ*xlnR*, Δ*araR*, Δ*clrA*, Δ*clrB* and Δ*xlnR*Δ*araR*Δ*clrA*Δ*clrB* mutant strains. Protein percentage values, which are < 0.1 are highlighted in red. Proteins, which show < 0.1% average value across all strains were excluded from analysis and are highlighted in grey. (B) Extracellular protein composition of control and mutant strains. Values represent the percentage of the total exoproteome.

**Data S3**. Extracellular proteome of *A. niger* control, Δ*xlnR*Δ*araR*Δ*clrA*Δ*clrB* and Δ*xlnR*Δ*araR*Δ*clrA*Δ*clrB*Δ*amyR* mutant strains. (A) Proteomics results of *A. niger* control, Δ*xlnR*Δ*araR*Δ*clrA*Δ*clrB* and Δ*xlnR*Δ*araR*Δ*clrA*Δ*clrB*Δ*amyR* mutant strains. Protein percentage values, which are < 0.1 are highlighted in red. Proteins, which show < 0.1% average value across all strains were excluded from analysis and are highlighted in grey. (B) Extracellular protein composition of control and mutant strains. Values represent the percentage of the total exoproteome.

**Fig. S1**. Relative contribution of XlnR, AraR, ClrA and ClrB towards utilization of wheat bran and related substrates. Contribution of each transcription factor is represented by the relative growth reduction of the corresponding single or multiple deletion mutants compared to the control. Relative growth has been estimated after 5, 6, 8, 10 and 14 days of incubation at 30°C. No growth difference was observed between biological replicates
**Fig. S2**. Relative contribution of AmyR towards utilization of maltose, starch and wheat bran. Contribution towards utilization of each substrate is represented by the relative growth reduction of the Δ*amyR* strain compared to the control, as well as the growth reduction of Δ*xlnR*Δ*araR*Δ*clrA*Δ*clrB*Δ*amyR* compared to Δ*xlnR*Δ*araR*Δ*clrA*Δ*clrB*. Relative growth has been estimated after 6, 8 and 10 days of incubation at 30°C. No growth difference was observed between biological replicates
**Fig. S3**. α‐glucosidase (AGD) and glucoamylase (GLA) activity of control, Δ*xlnR*Δ*araR*Δ*clrA*Δ*clrB* and Δ*xlnR*Δ*araR*Δ*clrA*Δ*clrB*Δ*amyR* mutant strains. Data represents the normalized mean values of biological duplicates and technical triplicates and the standard deviation. Letters (a‐c) are shown to explain the statistical differences between samples within each specific enzyme assay. Samples showing different letters show significant differences among the strains within each specific enzyme assay (ANOVA and Tukey's HDS test, *P* < 0.05)
**Table S1**. Sugar composition of the wheat bran used in this study. The analysis was performed as described previously for other plant biomass substrates.
**Table S2**. Binding site analysis of analysed CAZymes. The position of the binding site is specified with respect to the transcription start codon. The orientation of binding sites is represented by F (forward strand) or R (reverse strand).
**Table S3**. Putative intracellular proteins found in WB liquid culture supernatants. Prediction of secretion was performed based on WoLF PSORT and Phobius protein localization and signal peptide prediction tools. Values represent the percentage of the total extracellular proteome. The proteins which were not detected in the samples are marked in grey cells.
**Table S4**. *Aspergillus niger* strains used in this study.
**Table S5**. Primers used in this study. Homology flanks are highlighted in red.
**Table S6**. Summary of the ANOVA analysis for each enzymatic assay.
**Table S7**. Enzyme abbreviations used in this study.

## Data Availability

The mass spectrometry proteomic data have been deposited to the ProteomeXchange Consortium via the PRIDE (Perez‐Riverol *et al*., 2019) partner repository with the dataset identifier PXD023338 and 10.6019/PXD023338 (http://www.ebi.ac.uk/pride/archive/projects/PXD023338; Username: reviewer_pxd023338@ebi.ac.uk). All other data are available in the main text or in the supplementary files.

## References

[mbt213835-bib-0001] Alazi, E. , Niu, J. , Kowalczyk, J.E. , Peng, M. , Aguilar Pontes, M.V. , Van Kan, J.A. , *et al*. (2016) The transcriptional activator GaaR of *Aspergillus niger* is required for release and utilization of D‐galacturonic acid from pectin. FEBS Lett 590: 1804–1815. 27174630 10.1002/1873-3468.12211 PMC5111758

[mbt213835-bib-0002] Álvarez, C. , Reyes‐Sosa, F.M. , and Díez, B. (2016) Enzymatic hydrolysis of biomass from wood. Microb Biotechnol 9: 149–156. 26833542 10.1111/1751-7915.12346 PMC4767290

[mbt213835-bib-0003] Amores, G.R. , Guazzaroni, M.E. , Arruda, L.M. , and Silva‐Rocha, R. (2016) Recent progress on systems and synthetic biology approaches to engineer fungi as microbial cell factories. Curr Genomics 17: 85–98. 27226765 10.2174/1389202917666151116212255 PMC4864837

[mbt213835-bib-0004] Battaglia, E. , Visser, L. , Nijssen, A. , van Veluw, G. , Wösten, H. , and de Vries, R. (2011) Analysis of regulation of pentose utilisation in *Aspergillus niger* reveals evolutionary adaptations in Eurotiales. Stud Mycol 69: 31–38. 21892241 10.3114/sim.2011.69.03 PMC3161754

[mbt213835-bib-0005] Battaglia, E. , Zhou, M. , and de Vries, R.P. (2014) The transcriptional activators AraR and XlnR from *Aspergillus niger* regulate expression of pentose catabolic and pentose phosphate pathway genes. Res Microbiol 165: 531–540. 25086261 10.1016/j.resmic.2014.07.013

[mbt213835-bib-0006] Benocci, T. , Aguilar‐Pontes, M.V. , Zhou, M. , Seiboth, B. , and de Vries, R.P. (2017) Regulators of plant biomass degradation in ascomycetous fungi. Biotechnol Biofuels 10: 152. 28616076 10.1186/s13068-017-0841-x PMC5468973

[mbt213835-bib-0007] Benoit, I. , Culleton, H. , Zhou, M. , DiFalco, M. , Aguilar‐Osorio, G. , Battaglia, E. , *et al*. (2015) Closely related fungi employ diverse enzymatic strategies to degrade plant biomass. Biotechnol Biofuels 8: 107. 26236396 10.1186/s13068-015-0285-0 PMC4522099

[mbt213835-bib-0008] Budak, S. , Zhou, M. , Brouwer, C. , Wiebenga, A.d. , Benoit, I. , Di Falco, M. , *et al*. (2014) A genomic survey of proteases in Aspergilli. BMC Genom 15: 523. 10.1186/1471-2164-15-523 PMC4102723 24965873

[mbt213835-bib-0009] Chroumpi, T. , Peng, M. , Markillie, L.M. , Mitchell, H.D. , Nicora, C.D. , Hutchinson, C.M. , *et al*. (2021) Re‐routing of sugar catabolism provides a better insight into fungal flexibility in using plant biomass‐derived monomers as substrates. Front Bioeng Biotechnol 9: 167. 10.3389/fbioe.2021.644216 PMC7982397 33763411

[mbt213835-bib-0010] Coconi Linares, N. , Di Falco, M. , Benoit‐Gelber, I. , Gruben, B.S. , Peng, M. , Tsang, A. , *et al*. (2019) The presence of trace components significantly broadens the molecular response of *Aspergillus niger* to guar gum. N Biotechnol 51: 57–66. 30797054 10.1016/j.nbt.2019.02.005

[mbt213835-bib-0011] Coradetti, S.T. , Craig, J.P. , Xiong, Y. , Shock, T. , Tian, C. , and Glass, N.L. (2012) Conserved and essential transcription factors for cellulase gene expression in ascomycete fungi. Proc Nat Acad Sci USA 109: 7397–7402. 22532664 10.1073/pnas.1200785109 PMC3358856

[mbt213835-bib-0012] Coradetti, S.T. , Xiong, Y. , and Glass, N.L. (2013) Analysis of a conserved cellulase transcriptional regulator reveals inducer‐independent production of cellulolytic enzymes in *Neurospora crassa* . Microbiologyopen 2: 595–609. 23766336 10.1002/mbo3.94 PMC3948607

[mbt213835-bib-0013] Coutinho, P.M. , Andersen, M.R. , Kolenova, K. , vanKuyk, P.A. , Benoit, I. , Gruben, B.S. , *et al*. (2009) Post‐genomic insights into the plant polysaccharide degradation potential of *Aspergillus nidulans* and comparison to *Aspergillus niger* and *Aspergillus oryzae* . Fungal Genet Biol 46: S161–S169. 19618505 10.1016/j.fgb.2008.07.020

[mbt213835-bib-0014] de Vries, R.P. , Riley, R. , Wiebenga, A.d. , Aguilar‐Osorio, G. , Amillis, S. , Uchima, C.A. , *et al*. (2017) Comparative genomics reveals high biological diversity and specific adaptations in the industrially and medically important fungal genus *Aspergillus* . Genome Biol 18: 17‐1151. 28196534 10.1186/s13059-017-1151-0 PMC5307856

[mbt213835-bib-0015] Doench, J.G. , Hartenian, E. , Graham, D.B. , Tothova, Z. , Hegde, M. , Smith, I. , *et al*. (2014) Rational design of highly active sgRNAs for CRISPR‐Cas9–mediated gene inactivation. Nat Biotechnol 32: 1262–1267. 25184501 10.1038/nbt.3026 PMC4262738

[mbt213835-bib-0016] DuPont, M.S. , and Selvendran, R.R. (1987) Hemicellulosic polymers from the cell walls of beeswing wheat bran: part I, polymers solubilised by alkali at 2. Carbohydr Res 163: 99–113.

[mbt213835-bib-0017] Fowler, T. , Berka, R.M. , and Ward, M. (1990) Regulation of the *glaA* gene of *Aspergillus niger* . Curr Genet 18: 537–545. 2076554 10.1007/BF00327025

[mbt213835-bib-0018] Fujii, T. , Fang, X. , Inoue, H. , Murakami, K. , and Sawayama, S. (2009) Enzymatic hydrolyzing performance of *Acremonium cellulolyticus* and *Trichoderma reesei* against three lignocellulosic materials. Biotechnol Biofuels 2: 1–8. 19796378 10.1186/1754-6834-2-24 PMC2761304

[mbt213835-bib-0019] Gems, D. , Johnstone, I.L. , and Clutterbuck, A.J. (1991) An autonomously replicating plasmid transforms *Aspergillus nidulans* at high frequency. Gene 98: 61–67. 2013411 10.1016/0378-1119(91)90104-j

[mbt213835-bib-0020] Gielkens, M.M. , Dekkers, E. , Visser, J. , and de Graaff, L.H. (1999) Two cellobiohydrolase‐encoding genes from *Aspergillus niger* require D‐xylose and the xylanolytic transcriptional activator XlnR for their expression. Appl Environ Microbiol 65: 4340–4345. 10508057 10.1128/aem.65.10.4340-4345.1999 PMC91575

[mbt213835-bib-0021] Gomi, K. , Akeno, T. , Minetoki, T. , Ozeki, K. , Kumagai, C. , Okazaki, N. , and Iimura, Y. (2000) Molecular cloning and characterization of a transcriptional activator gene, *amyR*, involved in the amylolytic gene expression in *Aspergillus oryzae* . Biosci Biotechnol Biochem 64: 816–827. 10830498 10.1271/bbb.64.816

[mbt213835-bib-0022] Gruben, B.S. , Mäkelä, M.R. , Kowalczyk, J.E. , Zhou, M. , Benoit‐Gelber, I. , and De Vries, R.P. (2017) Expression‐based clustering of CAZyme‐encoding genes of *Aspergillus niger* . BMC Genom 18: 900. 10.1186/s12864-017-4164-x PMC5701360 29169319

[mbt213835-bib-0023] Gruben, B.S. , Zhou, M. , Wiebenga, A. , Ballering, J. , Overkamp, K.M. , Punt, P.J. , and De Vries, R.P. (2014) *Aspergillus niger* RhaR, a regulator involved in L‐rhamnose release and catabolism. Appl Microbiol Biotechnol 98: 5531–5540. 24682478 10.1007/s00253-014-5607-9

[mbt213835-bib-0024] Huang, L. , Dong, L. , Wang, B. , and Pan, L. (2020) The transcription factor PrtT and its target protease profiles in *Aspergillus niger* are negatively regulated by carbon sources. Biotechnol Lett 42: 613–624. 31970554 10.1007/s10529-020-02806-3

[mbt213835-bib-0025] Ishikawa, K. , Kunitake, E. , Kawase, T. , Atsumi, M. , Noguchi, Y. , Ishikawa, S. , *et al*. (2018) Comparison of the paralogous transcription factors AraR and XlnR in *Aspergillus oryzae* . Curr Genet 64: 1245–1260. 29654355 10.1007/s00294-018-0837-5

[mbt213835-bib-0026] Klaubauf, S. , Narang, H.M. , Post, H. , Zhou, M. , Brunner, K. , Mach‐Aigner, A.R. , *et al*. (2014) Similar is not the same: Differences in the function of the (hemi‐)cellulolytic regulator XlnR (Xlr1/Xyr1) in filamentous fungi. Fungal Genet Biol 72: 73–81. 25064064 10.1016/j.fgb.2014.07.007

[mbt213835-bib-0027] Kolbusz, M.A. , Di Falco, M. , Ishmael, N. , Marqueteau, S. , Moisan, M.‐C. , Baptista, C.d.S. , *et al*. (2014) Transcriptome and exoproteome analysis of utilization of plant‐derived biomass by *Myceliophthora thermophila* . Fungal Genet Biol 72: 10–20. 24881579 10.1016/j.fgb.2014.05.006

[mbt213835-bib-0028] Kowalczyk, J.E. , Gruben, B.S. , Battaglia, E. , Wiebenga, A. , Majoor, E. , and de Vries, R.P. (2015) Genetic interaction of *Aspergillus nidulans* galR, xlnR and araR in regulating D‐galactose and L‐arabinose release and catabolism gene expression. PLoS One 10: e0143200. 26580075 10.1371/journal.pone.0143200 PMC4651341

[mbt213835-bib-0029] Kowalczyk, J.E. , Khosravi, C. , Purvine, S. , Dohnalkova, A. , Chrisler, W.B. , Orr, G. , *et al*. (2017a) High resolution visualization and exo‐proteomics reveal the physiological role of XlnR and AraR in plant biomass colonization and degradation by *Aspergillus niger* . Environ Microbiol 19: 4587–4598. 29027734 10.1111/1462-2920.13923

[mbt213835-bib-0030] Kowalczyk, J.E. , Lubbers, R.J. , Peng, M. , Battaglia, E. , Visser, J. , and de Vries, R.P. (2017b) Combinatorial control of gene expression in *Aspergillus niger* grown on sugar beet pectin. Sci Rep 7: 1–12. 28955038 10.1038/s41598-017-12362-y PMC5617896

[mbt213835-bib-0031] Kun, R.S. , Meng, J. , Salazar‐Cerezo, S. , Mäkelä, M.R. , de Vries, R.P. , and Garrigues, S. (2020) CRISPR/Cas9 facilitates rapid generation of constitutive forms of transcription factors in *Aspergillus niger* through specific on‐site genomic mutations resulting in increased saccharification of plant biomass. Enzyme Microb Technol 136: 109508. 32331715 10.1016/j.enzmictec.2020.109508

[mbt213835-bib-0032] Li, N. , Kunitake, E. , Aoyama, M. , Ogawa, M. , Kanamaru, K. , Kimura, M. , *et al*. (2016) McmA‐dependent and ‐independent regulatory systems governing expression of ClrB‐regulated cellulase and hemicellulase genes in *Aspergillus nidulans* . Mol Microbiol 102: 810–826. 27588830 10.1111/mmi.13493

[mbt213835-bib-0033] Liaud, N. , Rosso, M.‐N. , Fabre, N. , Crapart, S. , Herpoël‐Gimbert, I. , Sigoillot, J.‐C. , *et al*. (2015) L‐lactic acid production by *Aspergillus brasiliensis* overexpressing the heterologous *ldha* gene from *Rhizopus oryzae* . Microb Cell Fact 14: 66. 25935554 10.1186/s12934-015-0249-x PMC4425913

[mbt213835-bib-0034] Mäkelä, M.R. , Hildén, K.S. , and de Vries, R.P. (2014) Chapter 8. Degradation and modification of plant biomass by fungi. In Fungal Genomics. The Mycota (A Comprehensive Treatise on Fungi as Experimental Systems for Basic and Applied Research) (vol. 13). Nowrousian M. (ed) Berlin, Heidelberg: Springer, pp. 175–208. 10.1007/978-3-642-45218-5_8.

[mbt213835-bib-0035] Meyer, V. , Arentshorst, M. , El‐Ghezal, A. , Drews, A.‐C. , Kooistra, R. , van den Hondel, C.A.M.J.J. , and Ram, A.F.J. (2007) Highly efficient gene targeting in the *Aspergillus niger kusA* mutant. J Biotechnol 128: 770–775. 17275117 10.1016/j.jbiotec.2006.12.021

[mbt213835-bib-0036] Morrison, W.R. (1978) Wheat lipid composition. Cereal Chem 55: 548–558.

[mbt213835-bib-0037] Nitsche, B.M. , Jørgensen, T.R. , Akeroyd, M. , Meyer, V. , and Ram, A.F.J. (2012) The carbon starvation response of *Aspergillus niger* during submerged cultivation: insights from the transcriptome and secretome. BMC Genom 13: 380. 10.1186/1471-2164-13-380 PMC3527191 22873931

[mbt213835-bib-0038] Nyombaire, G. (2012) Extrusion of wheat washed bran: physicochemical and functional properties. East Lansing: Michigan State University.

[mbt213835-bib-0039] Ogawa, M. , Kobayashi, T. , and Koyama, Y. (2012) ManR, a novel Zn (II) 2Cys6 transcriptional activator, controls the β‐mannan utilization system in *Aspergillus oryzae* . Fungal Genet Biol 49: 987–995. 23063954 10.1016/j.fgb.2012.09.006

[mbt213835-bib-0040] Ogawa, M. , Kobayashi, T. , and Koyama, Y. (2013) ManR, a transcriptional regulator of the β‐mannan utilization system, controls the cellulose utilization system in *Aspergillus oryzae* . Biosci Biotechnol Biochem 77: 426–429. 23391935 10.1271/bbb.120795

[mbt213835-bib-0041] Parker, M.L. , Ng, A. , and Waldron, K.W. (2005) The phenolic acid and polysaccharide composition of cell walls of bran layers of mature wheat (*Triticum aestivum* L. cv. Avalon) grains. J Sci Food Agric 85: 2539–2547.

[mbt213835-bib-0042] van Peij, N.N. , Gielkens, M.M. , de Vries, R.P. , Visser, J. , and de Graaff, L.H. (1998a) The transcriptional activator XlnR regulates both xylanolytic and endoglucanase gene expression in *Aspergillus niger* . Appl Environ Microbiol 64: 3615–3619. 9758775 10.1128/aem.64.10.3615-3619.1998 PMC106473

[mbt213835-bib-0043] van Peij, N.N. , Visser, J. , and De Graaff, L.H. (1998b) Isolation and analysis of *xlnR*, encoding a transcriptional activator co‐ordinating xylanolytic expression in *Aspergillus niger* . Mol Microbiol 27: 131–142. 9466262 10.1046/j.1365-2958.1998.00666.x

[mbt213835-bib-0044] Perez‐Riverol, Y. , Csordas, A. , Bai, J. , Bernal‐Llinares, M. , Hewapathirana, S. , Kundu, D.J. , *et al*. (2019) The PRIDE database and related tools and resources in 2019: improving support for quantification data. Nucleic Acids Res 47: D442–D450. 30395289 10.1093/nar/gky1106 PMC6323896

[mbt213835-bib-0045] Petersen, K. , Lehmbeck, J. , and Christensen, T. (1999) A new transcriptional activator for amylase genes in *Aspergillus* . Mol Gen Genet 262: 668–676. 10628849 10.1007/s004380051129

[mbt213835-bib-0046] Punt, P.J. , Schuren, F.H. , Lehmbeck, J. , Christensen, T. , Hjort, C. , and van den Hondel, C.A. (2008) Characterization of the *Aspergillus niger prtT*, a unique regulator of extracellular protease encoding genes. Fungal Genet Biol 45: 1591–1599. 18930158 10.1016/j.fgb.2008.09.007

[mbt213835-bib-0047] Raulo, R. , Kokolski, M. , and Archer, D.B. (2016) The roles of the zinc finger transcription factors XlnR, ClrA and ClrB in the breakdown of lignocellulose by *Aspergillus niger* . AMB Express 6: 5. 26780227 10.1186/s13568-016-0177-0 PMC4715039

[mbt213835-bib-0048] Rudjito, R.C. , Ruthes, A.C. , Jiménez‐Quero, A. , and Vilaplana, F. (2019) Feruloylated arabinoxylans from wheat bran: optimization of extraction process and validation at pilot scale. ACS Sustain Chem Eng 7: 13167–13177.

[mbt213835-bib-0049] Ruthes, A.C. , Martínez‐Abad, A. , Tan, H.‐T. , Bulone, V. , and Vilaplana, F. (2017) Sequential fractionation of feruloylated hemicelluloses and oligosaccharides from wheat bran using subcritical water and xylanolytic enzymes. Green Chem 19: 1919–1931.

[mbt213835-bib-0050] Song, L. , Ouedraogo, J.‐P. , Kolbusz, M. , Nguyen, T.T.M. , and Tsang, A. (2018) Efficient genome editing using tRNA promoter‐driven CRISPR/Cas9 gRNA in *Aspergillus niger* . PLoS One 13: e0202868. 30142205 10.1371/journal.pone.0202868 PMC6108506

[mbt213835-bib-0051] de Souza, W.R. , Maitan‐Alfenas, G.P. , de Gouvêa, P.F. , Brown, N.A. , Savoldi, M. , Battaglia, E. , *et al*. (2013) The influence of *Aspergillus niger* transcription factors AraR and XlnR in the gene expression during growth in D‐xylose, D‐arabinose and steam‐exploded sugarcane bagasse. Fungal Genet Biol 60: 29–45. 23892063 10.1016/j.fgb.2013.07.007

[mbt213835-bib-0052] Stevens, B.J. , and Selvendran, R.R. (1988) Changes in composition and structure of wheat bran resulting from the action of human faecal bacteria in vitro. Carbohydr Res 183: 311–319. 2850867 10.1016/0008-6215(88)84083-7

[mbt213835-bib-0053] Thomas‐Chollier, M. , Sand, O. , Turatsinze, J.‐v. , Janky, R. , Defrance, M. , Vervisch, E. , *et al*. (2008) RSAT: regulatory sequence analysis tools. Nucleic Acids Res 36: W119–W127. 18495751 10.1093/nar/gkn304 PMC2447775

[mbt213835-bib-0054] vanKuyk, P.A. , Benen, J.A.E. , Wösten, H.A.B. , Visser, J. , and de Vries, R.P. (2012) A broader role for AmyR in *Aspergillus niger*: regulation of the utilisation of D‐glucose or D‐galactose containing oligo‐ and polysaccharides. Appl Microbiol Biotechnol 93: 285–293. 21874276 10.1007/s00253-011-3550-6 PMC3251782

[mbt213835-bib-0055] de Vries, R.P. , van den Broeck, H.C. , Dekkers, E. , Manzanares, P. , de Graaff, L.H. , and Visser, J. (1999) Differential expression of three α‐galactosidase genes and a single β‐galactosidase gene from *Aspergillus niger* . Appl Environ Microbiol 65: 2453–2460. 10347026 10.1128/aem.65.6.2453-2460.1999 PMC91361

[mbt213835-bib-0056] de Vries, R.P. , Burgers, K. , van de Vondervoort, P.J.I. , Frisvad, J.C. , Samson, R.A. , and Visser, J. (2004) A new black *Aspergillus* species, *A. vadensis*, is a promising host for homologous and heterologous protein production. Appl Environ Microbiol 70: 3954–3959. 15240269 10.1128/AEM.70.7.3954-3959.2004 PMC444756

[mbt213835-bib-0057] de Vries, R.P. , and Visser, J. (1999) Regulation of the feruloyl esterase (*faeA*) gene from *Aspergillus niger* . Appl Environ Microbiol 65: 5500–5503. 10584009 10.1128/aem.65.12.5500-5503.1999 PMC91749

[mbt213835-bib-0058] de Vries, R.P. , and Visser, J. (2001) *Aspergillus* enzymes involved in degradation of plant cell wall polysaccharides. Microbiol Mol Biol Rev 65: 497–522. 11729262 10.1128/MMBR.65.4.497-522.2001 PMC99039

[mbt213835-bib-0059] de Vries, R. , van de Vondervoort, P. , Hendriks, L. , Van de Belt, M. , and Visser, J. (2002) Regulation of the α‐glucuronidase‐encoding gene (*aguA*) from *Aspergillus niger* . Mol Genet Genomics 268: 96–102. 12242504 10.1007/s00438-002-0729-7

[mbt213835-bib-0060] Yao, G. , Li, Z. , Gao, L. , Wu, R. , Kan, Q. , Liu, G. , and Qu, Y. (2015) Redesigning the regulatory pathway to enhance cellulase production in *Penicillium oxalicum* . Biotechnol Biofuels 8: 71. 25949521 10.1186/s13068-015-0253-8 PMC4422585

[mbt213835-bib-0061] Yuan, X.‐L. , Roubos, J.A. , Van Den Hondel, C.A. , and Ram, A.F. (2008a) Identification of InuR, a new Zn (II) 2Cys6 transcriptional activator involved in the regulation of inulinolytic genes in *Aspergillus niger* . Mol Genet Genomics 279: 11–26. 17917744 10.1007/s00438-007-0290-5 PMC2129107

[mbt213835-bib-0062] Yuan, X.‐L. , van der Kaaij, R.M. , van den Hondel, C.A. , Punt, P.J. , van der Maarel, M.J. , Dijkhuizen, L. , and Ram, A.F. (2008b) *Aspergillus niger* genome‐wide analysis reveals a large number of novel alpha‐glucan acting enzymes with unexpected expression profiles. Mol Genet Genomics 279: 545–561. 18320228 10.1007/s00438-008-0332-7 PMC2413074

