# Supplementary material for: Blocking utilization of major plant biomass polysaccharides leads Aspergillus niger towards utilization of minor components
Source: Microb Biotechnol. 2021 Jun 11;14(4):1683–98. doi: 10.1111/1751-7915.13835 (PMC8313289; doi:10.1111/1751-7915.13835)
Supplement: Supplementary file 4 — Fig. S1. Relative contribution of XlnR, AraR, ClrA and ClrB towards utilization of wheat bran and related substrates. Contribution of each transcription factor is represented by the relative growth reduction of the corresponding single or multiple deletion mutants compared to the control. Relative growth has been estimated after 5, 6, 8, 10 and 14 days of incubation at 30°C. No growth difference was observed between biological replicates Fig. S2. Relative contribution of AmyR towards utilization of maltose, starch and wheat bran. Contribution towards utilization of each substrate is represented by the relative growth reduction of the ΔamyR strain compared to the control, as well as the growth reduction of ΔxlnRΔaraRΔclrAΔclrBΔamyR compared to ΔxlnRΔaraRΔclrAΔclrB. Relative growth has been estimated after 6, 8 and 10 days of incubation at 30°C. No growth difference was observed between biological replicates Fig. S3. α‐glucosidase (AGD) and glucoamylase (GLA) activity of control, ΔxlnRΔaraRΔclrAΔclrB and ΔxlnRΔaraRΔclrAΔclrBΔamyR mutant strains. Data represents the normalized mean values of biological duplicates and technical triplicates and the standard deviation. Letters (a‐c) are shown to explain the statistical differences between samples within each specific enzyme assay. Samples showing different letters show significant differences among the strains within each specific enzyme assay (ANOVA and Tukey's HDS test, P < 0.05) Table S1. Sugar composition of the wheat bran used in this study. The analysis was performed as described previously for other plant biomass substrates. Table S2. Binding site analysis of analysed CAZymes. The position of the binding site is specified with respect to the transcription start codon. The orientation of binding sites is represented by F (forward strand) or R (reverse strand). Table S3. Putative intracellular proteins found in WB liquid culture supernatants. Prediction of secretion was performed based on WoLF PSORT and Phobius prote [file MBT2-14-1683-s001.pdf]

**Supplementary information**

## Title

Blocking utilization of major plant biomass polysaccharides leads *Aspergillus niger* towards utilization of minor components


**Authors**

Roland S. Kun[1], Sandra Garrigues[1], Marcos Di Falco[2], Adrian Tsang[2] & Ronald P. de Vries[1]

**Affiliations**

[1]Fungal Physiology, Westerdijk Fungal Biodiversity Institute & Fungal Molecular Physiology, Utrecht University, Uppsalalaan 8, 3584 CT Utrecht, The Netherlands.

[2]Centre for Structural and Functional Genomics, Concordia University, 7141 Sherbrooke Street West, Montreal, Quebec H4B 1R6, Canada.

**Corresponding author**

Ronald P. de Vries, E-mail: r.devries@wi.knaw.nl; Tel. +31 (0)30 21 22 600; Fax +31 (0)30 21 22 601.


## Table of Contents


Figures S1-S3

Tables S1-S7

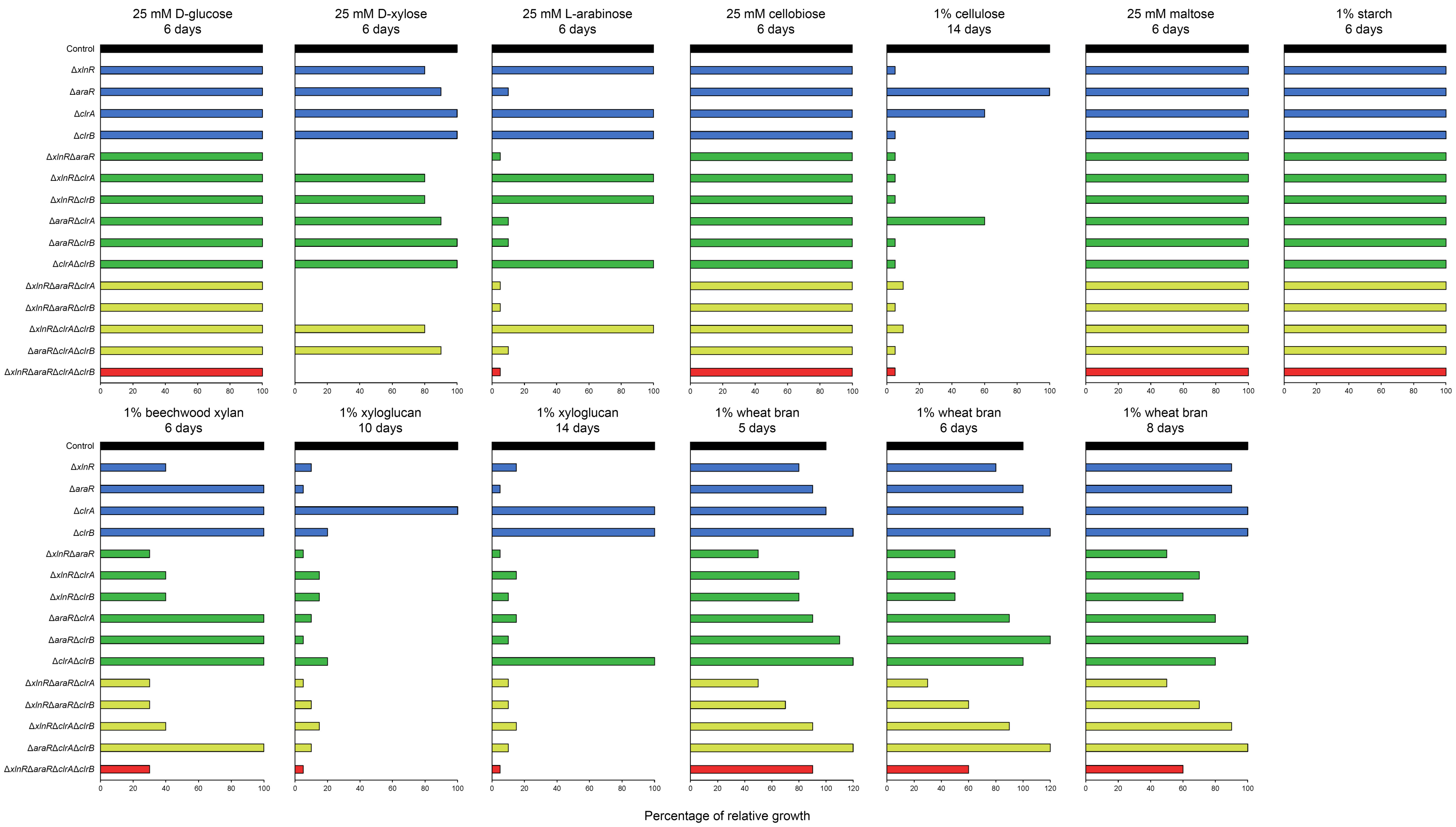


**Fig. S1.** Relative contribution of XlnR, AraR, ClrA and ClrB towards utilization of wheat bran and related substrates. Contribution of each transcription factor is represented by the relative growth reduction of the corresponding single or multiple deletion mutants compared to the control. Relative growth has been estimated after 5, 6, 8, 10 and 14 days of incubation at 30°C. No growth difference was observed between biological replicates.

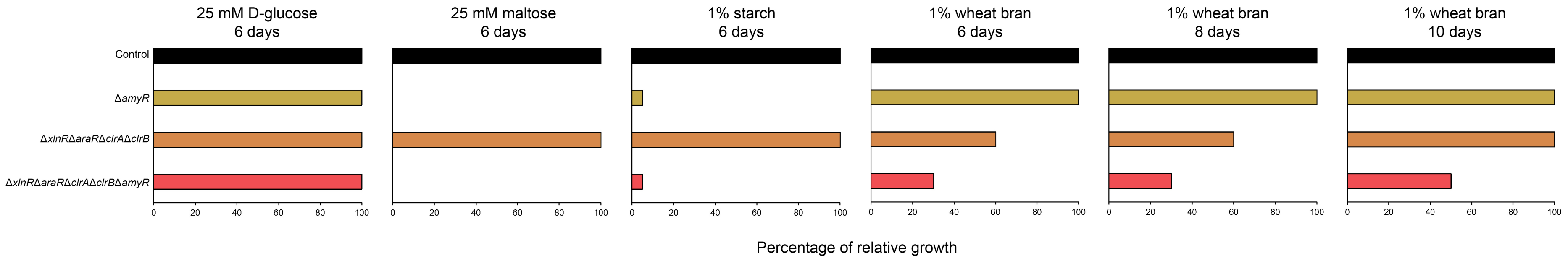


**Fig. S2.** Relative contribution of AmyR towards utilization of maltose, starch and wheat bran. Contribution towards utilization of each substrate is represented by the relative growth reduction of the Δ*amyR* strain compared to the control, as well as the growth reduction of Δ*xlnR*Δ*araR*Δ*clrA*Δ*clrB*Δ*amyR* compared to Δ*xlnR*Δ*araR*Δ*clrA*Δ*clrB* . Relative growth has been estimated after 6, 8 and 10 days of incubation at 30°C. No growth difference was observed between biological replicates.

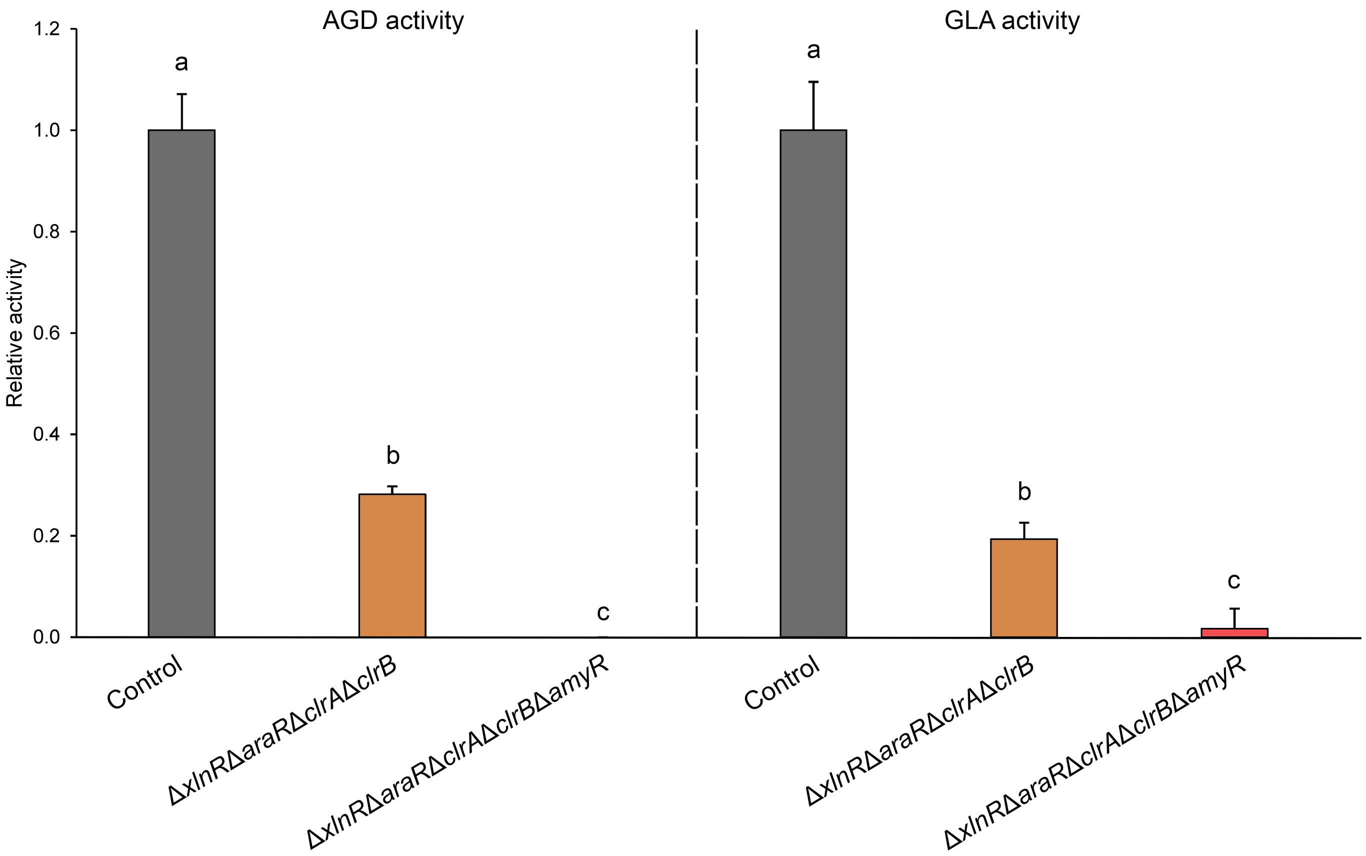


**Fig. S3.** α-glucosidase (AGD) and glucoamylase (GLA) activity of control, Δ*xlnR*Δ*araR*Δ*clrA*Δ*clrB* and Δ*xlnR*Δ*araR*Δ*clrA*Δ*clrB*Δ*amyR* mutant strains. Data represents the normalized mean values of biological duplicates and technical triplicates and the standard deviation. Letters (a-c) are shown to explain the statistical differences between samples within each specific enzyme assay. Samples showing different letters show significant differences among the strains within each specific enzyme assay (ANOVA and Tukey's HDS test, $p < 0.05$).

**Table S1.** Sugar composition of the wheat bran used in this study. The analysis was performed as described previously for other plant biomass substrates (Mäkelä *et al.* , 2017).

| mol% | | | | | w/w% |
|---|---|---|---|---|---|
| L-arabinose | D-xylose | D-mannose | D-galactose | D-glucose | Total sugar |
| 24.1 | 35.8 | 0.2 | 0.9 | 38.9 | 53.7 |

**Table S2.** Binding site analysis of analysed CAZymes. The position of the binding site is specified with respect to the transcription start codon. The orientation of binding sites is represented by F (forward strand) or R (reverse strand).

| Gene number | Gene name | XlnR | | AraR | ClrB | AmyR |
|---|---|---|---|---|---|---|
| | | GGCTAR (de Vries *et al.* , 2002) | CGGNTAAW (Ishikawa *et al.* , 2018) | CGGDTAAW (Ishikawa *et al.* , 2018) | CGGN8CCG (Li *et al.* , 2016) | CGGN8CGG (Petersen *et al.* , 1999) |
| NRRL3_08300 | *glaA* | R(617-612); R(549-544) | - | - | - | F(436-423); R(314-301) |
| NRRL3_09875 | *aamA* | R(651-646) | - | - | F/R(801-788) | - |
| NRRL3_08708 | *xlnC/xynA* | F(495-490); F(288-283) | - | - | - | F(900-887); R(983-970) |
| NRRL3_08707 | *axhA* | F(348-343); R(857-852) | F(19-12); R(200-193) | F(19-12); R(200-193) | - | F(377-364); R(460-447) |
| NRRL3_03768 | *abfB* | F(967-962); R(421-416); R(39-34) | F(842-835); R(250-243) | F(842-835); R(250-243) | F/R(740-727); F/R(447-434) | - |
| NRRL3_10449 | *bglA* | F(546-541) | - | - | - | - |
| NRRL3_00819 | *eglA* | F(710-705) | - | - | F/R(234-221) | R(464-451) |
| NRRL3_02451 | *xlnD/xynD* | F(148-143); F(134-129) | F(149-142); R(251-244) | R(251-244) | - | F(108-95); R(98-85) |
| NRRL3_00007 | *faeA* | F(265-260); F(229-224) | R(725-718) | R(725-718) | - | F(453-440) |
| NRRL3_05358 | *aglB* | F(645-640); R(643-638) | F(625-618) | F(625-618) | - | - |
| NRRL3_02630 | *lacA* | F(192-187); R(473-468) | R(474-467) | - | F/R(426-413) | F(855-842); R(741-728) |
| NRRL3_02584 | *cbhB* | F(417-412); R(157-152) | R(478-471); R(158-151) | R(478-471) | F/R(172-159) | F(328-315); R(153-140) |
| NRRL3_04953 | *cbhA* | F(912-907); F(731-726); R(1000-995) | - | - | F/R(408-395) | F(200-187) |
| NRRL3_04916 | *haeA* | F(218-213); R(924-919) | F(219-212) | - | F/R(665-652) | R(430-417) |
| NRRL3_08912 | *manA* | R(949-944) | - | - | F/R(121-108) | - |
| NRRL3_09019 | *cbhC* | F(584-579) | - | - | F/R(957-944); F/R(157-144); F/R(134-121) | - |
| NRRL3_06379 | *haeD* | - | F(217-210); F(126-119); R(146-139) | F(217-210); F(126-119); R(146-139) | - | - |
| NRRL3_04917 | *eglC* | F(128-123) | R(255-248) | R(255-248) | F/R(290-277) | - |
| NRRL3_10870 | *cbhD* | R(170-165) | R(359-352); R(171-164) | R(359-352) | - | - |
| NRRL3_01606 | *abfA* | - | - | - | - | - |
| NRRL3_00092 | *abnA* | - | - | - | F/R(166-153) | - |
| NRRL3_11773 | *gbgA* | - | - | - | F/R(400-387) | - |
| NRRL3_11747 | *aglA* | R(761-756) | R(825-818) | R(825-818) | - | - |
| NRRL3_07469 | *xghA* | F(765-760); F(77-72); R(299-294) | R(494-487); R(300-293) | R(494-487) | F/R(261-248) | F(564-551); R(597-584) |
| NRRL3_05252 | *pmeC* | R(525-520) | - | - | - | - |
| NRRL3_07470 | *pmeB* | F(657-652); R(879-874); R(191-186) | F(658-651); F(464-457) | F(464-457) | F/R(703-690) | F(367-354); R(400-387) |
| NRRL3_03087 | *inuE* | - | - | - | - | R(638-625); R(443-430) |
| NRRL3_08738 | *engA* | F(737-732); R(168-163) | - | - | - | F(855-842); R(856-843) |
| NRRL3_08701 | uncharacterized | - | F(757-750); F(365-358) | F(757-750); F(365-358) | F/R(639-626) | - |
| NRRL3_00743 | *aglD* | F(557-552) | F(151-144); F(100-93) | F(151-144); F(100-93) | - | - |

**Table S3.** Putative intracellular proteins found in WB liquid culture supernatants. Prediction of secretion was performed based on WoLF PSORT (Horton *et al.*, 2007) and Phobius (Käll *et al.*, 2004) protein localization and signal peptide prediction tools. Values represent the percentage of the total extracellular proteome. The proteins which were not detected in the samples are marked in grey cells.

| NRRL number | Description | First proteomics study | | | | | | Second proteomics study | | |
|---|---|---|---|---|---|---|---|---|---|---|
| | | Control | Δ*xlnR* | Δ*araR* | Δ*clrA* | Δ*clrB* | Δ*xlnR* Δ*araR* Δ*clrA* Δ*clrB* | Control | Δ*xlnR* Δ*araR* Δ*clrA* Δ*clrB* | Δ*xlnR* Δ*araR* Δ*clrA* Δ*clrB* Δ*amyR* |
| NRRL3_00054 | beta-1,3-glucanosyltransferase | 0.024 | 0.066 | 0.015 | 0.022 | 0.027 | 0.024 | 0.008 | 0.025 | 0.025 |
| NRRL3_05340 | glycoside hydrolase/deacetylase domain-containing protein | 0.016 | 0.003 | 0.022 | 0.007 | 0.018 | 0.007 | 1.23E-04 | 3.49E-05 | 1.63E-05 |
| NRRL3_06317 | beta-1,3-glucanosyltransferase | 0.011 | 0.002 | 0.019 | 0.005 | 0.017 | 0.004 | 0.012 | 0.003 | 0.006 |
| NRRL3_10021 | glycoside hydrolase family 16 protein | 0.007 | 0.007 | 0.009 | 0.010 | 0.010 | 0.015 | 0.002 | 0.004 | 0.005 |
| NRRL3_03448 | carboxylesterase | 0.001 | 0.007 | 3.80E-04 | 0.001 | 0.002 | 0.001 | 0.001 | 0.004 | 0.002 |
| NRRL3_06962 | hypothetical protein | 0.001 | 0.002 | 0.001 | 0.002 | 0.002 | 0.006 | 0 | 0 | 0 |
| NRRL3_05191 | carboxylesterase | 9.17E-05 | 2.73E-04 | 1.60E-04 | 6.87E-05 | 4.77E-04 | 0.001 | 0 | 0 | 0 |
| NRRL3_09188 | enamine/imine deaminase | 8.48E-05 | 1.90E-04 | 3.38E-04 | 0 | 0.001 | 0 | 0 | 0 | 0 |
| NRRL3_10668 | 14-3-3 protein | 0 | 0.001 | 1.05E-04 | 0 | 6.57E-05 | 0.003 | 0 | 0 | 0 |
| NRRL3_09732 | patched family protein | 0 | 0 | 0 | 0 | 0 | 0 | 0.002 | 0 | 0 |
| NRRL3_05710 | ubiquitin-40S ribosomal protein S27a | 0 | 0 | 0 | 0 | 0 | 0 | 0.001 | 0 | 0 |
| NRRL3_04179 | uncharacterized protein | 0 | 0 | 0 | 0 | 0 | 0 | 0.001 | 2.81E-03 | 0.003 |
| NRRL3_04048 | gamma interferon inducible lysosomal thiol reductase | 0 | 0 | 0 | 0 | 0 | 0 | 1.22E-04 | 0 | 0 |
| NRRL3_05712 | ribonuclease T2-like protein | 0 | 0 | 0 | 0 | 0 | 0 | 0 | 0 | 3.97E-04 |
| Total % of putative intracellular proteins | | 0.060 | 0.089 | 0.067 | 0.047 | 0.077 | 0.061 | 0.027 | 0.039 | 0.042 |

**Table S4.** *Aspergillus niger* strains used in this study.

| **Strain ID** | **CBS number** | **Reference** |
|---|---|---|
| N593 Δ*kusA* (Control strain) | CBS 138852 | Meyer *et al.*, 2007 |
| Δ*xlnR* | CBS 145447 | This study |
| Δ*araR* | CBS 145451 | This study |
| Δ*clrA* | CBS 145444 | This study |
| Δ*clrB* | CBS 145445 | This study |
| Δ*xlnR* Δ*araR* | CBS 145455 | This study |
| Δ*xlnR* Δ*clrA* | CBS 145448 | This study |
| Δ*xlnR* Δ*clrB* | CBS 145449 | This study |
| Δ*araR* Δ*clrA* | CBS 145452 | This study |
| Δ*araR* Δ*clrB* | CBS 145453 | This study |
| Δ*clrA* Δ*clrB* | CBS 145446 | This study |
| Δ*xlnR* Δ*araR* Δ*clrA* | CBS 145456 | This study |
| Δ*xlnR* Δ*araR* Δ*clrB* | CBS 145457 | This study |
| Δ*xlnR* Δ*clrA* Δ*clrB* | CBS 145450 | This study |
| Δ*araR* Δ*clrA* Δ*clrB* | CBS 145454 | This study |
| Δ*xlnR* Δ*araR* Δ*clrA* Δ*clrB* | CBS 145458 | This study |
| Δ*xlnR* Δ*araR* Δ*clrA* Δ*clrB* Δ*amyR* | CBS 146415 | This study |

**Table S5.** Primers used in this study. Homology flanks are highlighted in red.

| Primer name | Sequence | Description |
|---|---|---|
| **Primers for construction of repair templates** | | |
| *xlnR*-5F | GTGTGTGTGTGAGAGAGAAAGG | for amplification of 5' flank region of *xlnR* |
| *xlnR*-5R | CGATAGCGAATCCTAGCAGTGGAAAGTGAGGTATTCAGACCG | |
| *xlnR*-3F | ACTGCTAGGATTCGCTATCGACACGGCTGATGATGAGATGC | for amplification of 3' flank region of *xlnR* |
| xlnR-3R | GACGAGAGGAGTTGGTAGCG | |
| *xlnR*-NEST-F | CTTTCTCGTGGGTTCTTCACC | for 5' + 3' flank fusion of *xlnR* repair template |
| *xlnR*-NEST-R | GGATGTAGTCGTCCAGGAGG | |
| *araR*-5F | GTCCGCAAGTTGTGTGGTGG | for amplification of 5' flank region of *araR* |
| *araR*-5R | CGATAGCGAATCCTAGCAGTGGAATCGCAGTCTGATGAAACG | |
| *araR*-3F | ACTGCTAGGATTCGCTATCGGTTTCTCACAGCACCGATGC | for amplification of 3' flank region of *araR* |
| *araR*-3R | AACCGAGAAGCCCAGTTTCG | |
| *araR*-NEST-F | GAAGCGACCTCATAGCGACC | for 5' + 3' flank fusion of *araR* repair template |
| *araR*-NEST-R | ATGCCAGAAACATGCGATGC | |
| *clrA*-5F | TCAGCGAAACCAGTAAGAACG | for amplification of 5' flank region of *clrA* |
| *clrA*-5R | CGATAGCGAATCCTAGCAGTGTTGTTGTGTTCAAGGGTGC | |
| *clrA*-3F | ACTGCTAGGATTCGCTATCGCATCATACGCATCTTGTCAGTCC | for amplification of 3' flank region of *clrA* |
| *clrA*-3R | GGCAGTATTAACCAGGCTTGC | |
| *clrA*-NEST-F | TCAGCGAAACCAGTAAGAACG | for 5' + 3' flank fusion of *clrA* repair template |
| *clrA*-NEST-R | GGCAGTATTAACCAGGCTTGC | |
| *clrB*-5F | ATCACACAACCCTTCTCGTACC | for amplification of 5' flank region of *clrB* |
| *clrB*-5R | CGATAGCGAATCCTAGCAGTTGCTGGAGTTTTTGTTTGACGG | |
| *clrB*-3F | ACTGCTAGGATTCGCTATCGCTGTACTTACAGGGTGCAGC | for amplification of 3' flank region of *clrB* |
| *clrB*-3R | GGATGGATCGTCTTAGGATGC | |
| *clrB*-NEST-F | ATCACACAACCCTTCTCGTACC | for 5' + 3' flank fusion of *clrB* repair template |
| *clrB*-NEST-R | GGATGGATCGTCTTAGGATGC | |
| *amyR*-5F | TGGGATGTTCACCAGTGTTACG | for amplification of 5' flank region of *amyR* |
| *amyR*-5R | CGATAGCGAATCCTAGCAGTGCGGAGACAAGTGTGACTCC | |
| *amyR*-3F | ACTGCTAGGATTCGCTATCGCAACTACGACGATGACGATGC | for amplification of 3' flank region of *amyR* |
| *amyR*-3R | CACCGTGACCCAGAGAAAGG | |
| *amyR*-NEST-F | GAGCCTCAGACTCTGTCAGC | for 5' + 3' flank fusion of *amyR* repair template |
| *amyR*-NEST-R | TCCACCACCATCAAAATCACC | |
| **Primers for CRISPR/Cas9 sgRNA construction** | | |
| P1-gRNA | CAACCTCCAATCCAATTTGACTCCGCCGAACGTACTG | 5F-sgRNA; for amplification of 5' flank region and fusion of sgRNA construct |
| P2-gRNA | ACTACTCTACCACTATTTGAAAAGCAAAAAAGGAAGGTACAAAAAAGC | 3R-sgRNA; for amplification of 3' flank region and fusion of sgRNA construct |
| P3-*xlnR* | CGGTCTCCTGGCGAGTATGCGACGAGCTTACTCGTTTCG | 5R-xlnR; for amplification of 5' flank region of sgRNA construct |
| P4-*xlnR* | GCATACTCGCCAGGAGACCGGTTTTAGAGCTAGAAATAGCAAG | 3F-xlnR; for amplification of 3' flank region of sgRNA construct |
| P3-*araR* | CCCAGAAAGTCAGGGCACACGACGAGCTTACTCGTTTCG | 5R-araR; for amplification of 5' flank region of sgRNA construct |
| P4-*araR* | GTGTGCCCTGACTTTCTGGGGTTTTAGAGCTAGAAATAGCAAG | 3F-araR; for amplification of 3' flank region of sgRNA construct |
| P3-*clrA* | TGGAAGAATTCGATGACAGGGACGAGCTTACTCGTTTCG | 5R-clrA; for amplification of 5' flank region of sgRNA construct |
| P4-*clrA* | CCTGTCATCGAATTCTTCCAGTTTTAGAGCTAGAAATAGCAAG | 3F-clrA; for amplification of 3' flank region of sgRNA construct |
| P3-*clrB* | TCCTTTCTTCAGACCTGAGCGACGAGCTTACTCGTTTCG | 5R-clrB; for amplification of 5' flank region of sgRNA construct |
| P4-*clrB* | GCTCAGGTCTGAAGAAAGGAGTTTTAGAGCTAGAAATAGCAAG | 3F-clrB; for amplification of 3' flank region of sgRNA construct |
| P3-*amyR* | TGCGATAAGTGTCGACGCCTGACGAGCTTACTCGTTTCG | 5R-amyR; for amplification of 5' flank region of sgRNA construct |
| P4-*amyR* | AGGCGTCGACACTTATCGCAGTTTTAGAGCTAGAAATAGCAAG | 3F-amyR; for amplification of 3' flank region of sgRNA construct |
| **Primers for screening transformant colonies** | | |
| Δlinker-F | ACTGCTAGGATTCGCTATCG | primers matching the linker in the repair templates |
| Δlinker-R | CGATAGCGAATCCTAGCAGT | |
| *xlnR*-F | GTCTCAGACTGTCGGGTTGG | for screening the presence of *xlnR* gene |
| *xlnR*-R | AAGTCCTCTGGGATGCGTCC | |
| Δ*xlnR*-5F | GAGATGCCTACGGAAGAGTCG | for screening the position of the repair template (together with Dlinker-R/Dlinker-F) |
| Δ*xlnR*-3R | TGTATGCTTGATCCCAAGTCG | |
| *araR*-F | GTCAGGGCACACTGTTAATGC | for screening the presence of *araR* gene |
| *araR*-R | CATCAAGAGCGTGAGAGATGG | |
| Δ*araR*-5F | GGGAAGAGATCCTTGAACAGG | for screening the position of the repair template (together with Dlinker-R/Dlinker-F) |
| Δ*araR*-3R | CCTCCGTAAAACTCCTCATCC | |
| *clrA*- F | TGTCAGTCTCGCCAACATACC | for screening the presence of *clrA* gene |
| *clrA*- R | AGTGTCAAGATGCTGTCTTTTTGG | |
| Δ*clrA*- 5F | GAGACATGGCACTGAAGTTCC | for screening the position of the repair template (together with Dlinker-R/Dlinker-F) |
| Δ*clrA*- 3R | CAAGTCGGCTCAAGTTCTCG | |
| Δ*clrA*-F | CATAGCAAAGCACTTTAGCCAGG | for screening the deletion of *clrA* |
| Δ*clrA*-R | CCATACTTGAAACTTCCTCTTCCC | |
| *clrB*- F | GTGTGACTGTCCCCTTCTTCC | for screening the presence of *clrB* gene |
| *clrB*- R | TTCCTTATGTAGCCCCAGAGC | |
| Δ*clrB*- 5F | TATCTCAAAAGCTCGGTGTGG | for screening the position of the repair template (together with Dlinker-R/Dlinker-F) |
| Δ*clrB*- 3R | GTAAGTGTCTCGTTCGTAGGG | |
| Δ*clrB*-F | TGCTCACTTCCCCAAAACAGG | for screening the deletion of *clrB* |
| Δ*clrB*-R | GTGTTCTCGACATAAATCCTCCG | |
| Δ*amyR*-F | TGGGATGTTCACCAGTGTTACG | for screening the deletion of *amyR* |
| Δ*amyR*-R | CACCGTGACCCAGAGAAAGG | |

**Table S6.** Summary of the ANOVA analysis for each enzymatic assay.

| Condition | N. of variables[a] | DF[b] | F-value | *p* -value[c] |
|---|---|---|---|---|
| ABF | 16 | 95 | 1339.78 | 0.0000 |
| AGL | 16 | 95 | 4122.87 | 0.0000 |
| BGL | 16 | 95 | 486.67 | 0.0000 |
| BXL | 16 | 95 | 1431.77 | 0.0000 |
| LAC | 16 | 95 | 442.95 | 0.0000 |
| AGD | 3 | 17 | 892.93 | 0.0000 |
| GLA | 3 | 17 | 420.49 | 0.0000 |

[a] Number of variables within each ANOVA analysis

[b] DF: Degrees of freedom

[c] *p*-values of the F-test. Statiscal significance is refered when $p < 0.05$

**Table S7.** Enzyme abbreviations used in this study.

| Abbreviation | Enzyme |
|---|---|
| ABF | α-arabinofuranosidase |
| ABN | endo-arabinanase |
| AE | acetyl esterase |
| AGL | α-1,4-galactosidase |
| AMY | α-amylase |
| AXH | arabinoxylan arabinofuranohydrolase |
| BGL | β-1,4-glucosidase |
| BGN | endo-1,6-β-D-glucanase |
| BXL | β-1,4-xylosidase |
| CBH | cellobiohydrolase |
| EGL | β-1,4-endo-glucanase |
| FAE | feruloyl esterase |
| GLA | glucoamylase |
| GLN | exo-1,6-galactanase |
| INX | exo-inulinase |
| LAC | β-1,4-galactosidase |
| MAN | β-1,4-endo-mannanase |
| PME | pectin methyl esterase |
| XG-EGL | xyloglucanase |
| XGH | xylogalacturonase |
| XLN | β-1,4-endo-xylanase |

**Supplementary Information References**

de Vries. R., van de Vondervoort, P., Hendriks, L., Van de Belt, M., and Visser, J. (2002) Regulation of the α-glucuronidase-encoding gene (*aguA*) from *Aspergillus niger*. *Mol Genet Genomics* **268**: 96-102.

Horton, P., Park, K.J., Obayashi, T., Fujita, N., Harada, H., Adams-Collier, C.J., and Nakai, K. (2007) WoLF PSORT: protein localization predictor. *Nucleic Acids Res* **35**: 585-587.

Ishikawa, K., Kunitake, E., Kawase, T., Atsumi, M., Noguchi, Y., Ishikawa, S., *et al.* (2018) Comparison of the paralogous transcription factors AraR and XlnR in *Aspergillus oryzae*. *Curr Genet* **64**: 1245-1260.

Käll, L., Krogh, A., and Sonnhammer, E.L.L.A. (2004) Combined transmembrane topology and signal peptide prediction method. *J Mol Biol* **338**: 1027-1036.

Li, N., Kunitake, E., Aoyama, M., Ogawa, M., Kanamaru, K., Kimura, M., *et al.* (2016) McmA-dependent and -independent regulatory systems governing expression of ClrB-regulated cellulase and hemicellulase genes in *Aspergillus nidulans*. *Mol Microbiol* **102**: 810-826.

Mäkelä, M.R., Bouzid, O., Robl, D., Post, H., Peng M, Heck, A., *et al.* (2017) Cultivation of *Podospora anserina* on soybean hulls results in an efficient enzyme cocktail for plant biomass hydrolysis. *New Biotechnol* **37**: 162-171.

Meyer, V., Arentshorst, M., El-Ghezal, A., Drews, A.C., Kooistra, R., van den Hondel, C.A.M.J.J., and Ram, A.F.J. (2007) Highly efficient gene targeting in the *Aspergillus niger kusA* mutant. *J Biotechnol* **128**: 770-775.

Petersen, K.L., Lehmbeck, J., and Christensen, T. (1999) A new transcriptional activator for amylase genes in *Aspergillus*. *Mol Gen Genet* **262**: 668-676.
